# Recent Progress in Energy‐Driven Water Splitting

**DOI:** 10.1002/advs.201600337

**Published:** 2017-01-13

**Authors:** Si Yin Tee, Khin Yin Win, Wee Siang Teo, Leng‐Duei Koh, Shuhua Liu, Choon Peng Teng, Ming‐Yong Han

**Affiliations:** ^1^ Institute of Materials Research and EngineeringAgency for ScienceTechnology and Research2 Fusionopolis WaySingapore138634; ^2^ Department of Biomedical EngineeringNational University of Singapore9 Engineering DriveSingapore117576; ^3^ School of Material Science and EngineeringNanyang Technological UniversitySingapore639798

**Keywords:** electrochemical water splitting, hydrogen generation, photoelectrochemical water splitting, photocatalytic water splitting, solar water splitting

## Abstract

Hydrogen is readily obtained from renewable and non‐renewable resources via water splitting by using thermal, electrical, photonic and biochemical energy. The major hydrogen production is generated from thermal energy through steam reforming/gasification of fossil fuel. As the commonly used non‐renewable resources will be depleted in the long run, there is great demand to utilize renewable energy resources for hydrogen production. Most of the renewable resources may be used to produce electricity for driving water splitting while challenges remain to improve cost‐effectiveness. As the most abundant energy resource, the direct conversion of solar energy to hydrogen is considered the most sustainable energy production method without causing pollutions to the environment. In overall, this review briefly summarizes thermolytic, electrolytic, photolytic and biolytic water splitting. It highlights photonic and electrical driven water splitting together with photovoltaic‐integrated solar‐driven water electrolysis.

## 1 Introduction

The world's population has surpassed seven billion and is estimated to reach more than nine billion by this mid‐century, which will account for more than doubled energy consumption.^1^ As such, there is an increased demand to develop renewable energy resources because the commonly used non‐renewable energy resources will be exhausted eventually. As the most abundant element in the universe, hydrogen is combusted to produce only water and thus it is regarded as a future promising renewable energy resource when it can be produced efficiently and cheaply. Also, hydrogen is more efficient than conventional energy sources to produce 2.5 times more energy per unit mass of fuel.^2^ Nowadays, it takes a substantial amount of non‐renewable energy to produce hydrogen at industrial scale for practical applications. It remains very challenging to achieve highly efficient production of hydrogen with the use of renewable energy.

Today, hydrogen production is readily generated from thermal energy at high temperature through steam reforming via reacting fossil fuel with steam (e.g. CH_4_ + H_2_O → CO + 3H_2_) or coal gasification via reacting fossil fuel in the presence of a controlled amount of oxygen and/or steam (e.g. 3C + O_2_ + H_2_O → H_2_ + 3CO). In the pursuit of carbon‐free energy, electrical energy is promisingly used to electrolyze water to hydrogen, which account for 4% of the world's hydrogen production as compared to more than 90% driven by thermal energy. Recently, there is an increased focus on photonic energy driven hydrogen production. With the most abundant solar and water resources on Earth, solar hydrogen production can acquire hydrogen directly from water splitting under sunlight to obtain the endless clean fuel for various applications. It is worthy to note that biochemical energy can also convert biomass to hydrogen by microorganisms via biological processes including dark‐fermentation, photo‐fermentation and biophotolysis. So far, thermal and electrical energy are the major non‐renewable energy for hydrogen production, which are dominantly produced by burning of fossil fuel. As fossil fuel will become scarce and expensive within lifetimes of humans, there will be an inevitable transition to renewable energy resources, which can generate thermal, electrical, photonic and biochemical energy for hydrogen production.

In this review, we first provide a brief summary on the various energy‐driven hydrogen production routes with the utilization of thermal, electrical, photonic and biochemical energy. Hydrogen production is not only limited to thermolysis, electrolysis, photolysis and biolysis of water, but also depends on thermoelectrolysis, photoelectrolysis and biophotolysis of water. To improve the efficiency of hydrogen generation, catalytic water splitting is emphasized in recent research including electrocatalysis, photocatalysis, thermochemical cycle and enzymatic reaction. Here, we pay more attention to highlight the new advances in catalyst fabrication and recently focused photonic and electrical driven water splitting. Overall, this is a timely review to summarize various renewable hydrogen production routes and also deliberate more on photovoltaic‐integrated solar driven water electrolysis.

## 2 Renewable Hydrogen Production

The world's energy resources can be categorized into fossil fuel, nuclear fuel and renewable ones. Renewable resources are available all year round, whereas non‐renewable resources will be depleted eventually. The overuse of the non‐renewable energy has caused serious effects to our environment due to the toxic gas emission (e.g. CO_x_,NO_x_, SO_x_, C_x_H_y_) and radioactive pollution.^3^ There are different forms of renewable energy resources including wind, water, sun and biomass, which can be used directly/indirectly to generate sustainable thermal, electrical, photonic and biochemical energy for hydrogen generation via water splitting reaction. Currently, thermal energy is still the major driving force to produce hydrogen dominantly through steam reforming of fossil fuel, compared to only 4% production through electrolysis using electrical energy. The pollution problem of fossil fuel and the finite nature of conventional fuels have forced the world to look for alternative renewable energy resources. From past progress, electricity can also be readily powered by natural renewable resources besides the non‐renewable fossil/nuclear fuel. The electrolysis of water is one of the most promising environmentally benign processes for future hydrogen production. The energy input to drive hydrogen production can also be retrieved from solar radiation. Meanwhile, biological hydrogen production can be achieved by microorganisms through dark or photo‐fermentation.^3^

## 3 Energy‐Driven Water Splitting

**Figure 1:**
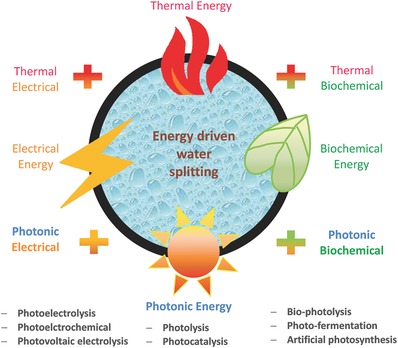
Various energy driven water splitting routes by using thermal, electrical, biochemical and photonic energy or their combinations.

Thermodynamically, water splitting is an uphill reaction rather than a spontaneous process that requires an external energy to drive it because a back reaction proceeds easily. Hydrogen production is readily obtained by using different forms of energy from renewable and non‐renewable resources. **Figure** **1** summarizes various water splitting routes that are driven by thermal, electrical, photonic and biochemical energy through thermolysis (e.g. thermochemical cycles), electrolysis (e.g. electrocatalysis), photolysis (e.g. photocatalysis) and biolysis (e.g. dark‐fermentation), respectively. Hydrogen production is also readily obtained by using two or more forms of energy. A number of important hybrid energy systems are defined as thermal + electrical (e.g. thermoelectrolysis, high‐temperature electrolysis), electrical + photonic (e.g. photoelectrolysis, photovoltaic electrolysis), and photonic + biochemical (e.g. biophotolysis, photo‐fermentation). In comparison with the non‐hybrid systems, the hybrid systems are generally more thermodynamically favorable as a part of the required energy is substituted with a cheaper or renewable resource, consequently lowering the overall operation cost and activation barrier and improving the chemical reaction kinetics and hydrogen production rate. Together with four non‐hybrid systems, four hybrid systems are also described in this review including thermoelectrolysis, biophotolysis, photoelectrolysis and photovoltaic‐integrated solar driven water electrolysis. With the recent development of advanced catalysts, more emerging electrocatalytic, photocatalytic, photoelectrochemical and photobiological water splitting have been extensively explored to produce hydrogen, which are described in this review as well. It is noted that nuclear energy is also used to dissociate water into hydrogen and hydroxyl radicals under nuclear (mainly alpha) radiation. These radicals are chemically reactive, and in turn recombine to produce a series of highly reactive combinations instead such as superoxide (HO_2_) and peroxide (H_2_O_2_).

### 3.1 Non‐Hybrids

*Thermal Energy*: Thermolysis of water involves the chemical dissociation of water into hydrogen and oxygen when thermal energy is applied at high temperature (>2500 K).^4^ As the dissociation of water is reversible, hydrogen and oxygen are required to be separated effectively so as to prevent their recombination back to water. As the thermolysis of water occurs at high temperature, intermediate substances (catalysts) are used in thermochemical cycle to greatly reduce the temperature (<1200 K) for water dissociation into hydrogen and oxygen.^4^, ^5^ The development of a practical thermochemical cycle for water splitting is still facing issues such as great complexity of reaction kinetics and continuous recovery of the intermediate substances.^5^

*Electrical Energy*: Electrolysis of water involves the chemical decomposition of water into hydrogen and oxygen at the respective electrodes when an electric current passes through water. There are three main types of electrolytic cells for water splitting including alkaline electrolysis cells, polymer electrolyte membrane (PEM) cells and solid oxide electrolysis cells (SOECs). In recent years, electrocatalytic water splitting becomes important for improving its efficiency and cost‐effectiveness.

*Photonic Energy*: Photolysis of water involves the chemical breakdown of water into hydrogen and oxygen by photonic energy. As the potential for water splitting process is 1.23 eV that corresponds to the light of 1008 nm, this indicates that the dissociation of water can theoretically reach down into infrared light. That is to say, ≈70% of the solar‐irradiated photons are eligible for driving water splitting. On the other hand, water itself does not absorb appreciable radiation in visible and near ultraviolet ranges. The dissociation of water is technically possible by exposing water to ultraviolet light, X‐rays or gamma rays to transduce the radiant energy to chemical energy. Today, this direct water splitting under the high‐frequency radiation holds less interest in industrial application since visible and infrared light are the major constituent of solar light. In recent years, the photocatalytic water splitting under the low‐frequency radiation becomes important for improving its efficiency and cost‐effectiveness.

*Biochemical Energy*: Fermentative conversion of carbohydrates to hydrogen can be manifested in dark by a diverse group of anaerobic bacteria. The dark‐fermentation can produce hydrogen through an anaerobic process in the absence of oxygen (i.e. C_6_H_12_O_6_ + 2H_2_O → 2CH_3_COOH + 2CO_2_ + 4H_2_). This fermentative production of hydrogen takes place through enzymatic hydrolysis of high molecular weight organics to water‐soluble organics followed by the production of hydrogen together with carbon dioxide and fatty acids.^3^

### 3.2 Hybrids

*Thermoelectrolysis*: Thermoelectrolysis of water involves the chemical dissociation of water with the combined use of electrical and thermal energy. It is more efficient and economic at high temperature because a substantial part of the required energy is supplied with cheaper thermal energy, and this considerably reduces the demand of electrical energy and accelerates the electrolytic reaction kinetics at high temperature.^6^ Alkaline electrolysis optimally operates at high temperature of near 200 °C and is used for hydrogen production at industrial scale. PEM electrolyzers typically operate below 100 °C (more efficient than alkaline electrolysis) and become increasingly available for commercial application. SOEC electrolyzers are the most electrically efficient but the least developed. SOEC technology faces challenges with fast material degradation and limited long term stability.^7^

*Biophotolysis*: Biophotolysis of water involves oxygenic photosynthesis by microorganisms (i.e. green microalgae and cyanobacteria) with the combined use of biochemical and photonic energy for hydrogen production via direct and indirect method. In direct biophotolysis, when microorganisms split water into hydrogen ion and oxygen through capturing solar light, the generated hydrogen ions are further converted into hydrogen by hydrogenase enzyme (i.e. 2H_2_O + solar → 2H_2_ + O_2_). In indirect biophotolysis, solar energy is captured by microorganisms via photosynthesis and stored in some form of carbohydrate (e.g. 6CO_2_ + 12H_2_O + solar → C_6_H_12_O_6_ + 6O_2_), which is later used to produce hydrogen (e.g. C_6_H_12_O_6_ + 12H_2_O + solar → 12H_2_ + 6CO_2_). There is another biophotolytic process, photo‐fermentation, which involves the capture and conversion of solar energy using photosynthetic bacteria to degrade carbohydrate (organic aids as electron donors) into hydrogen and carbon dioxide (e.g. CH_3_COOH + 2H_2_O + solar → 4H_2_ + 2CO_2_).

## 4 Photocatalytic Water Splitting

**Figure 2:**
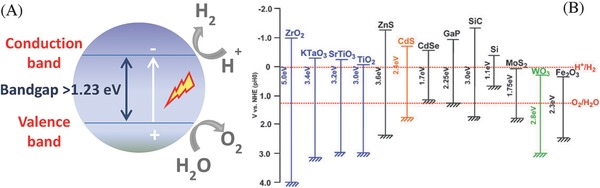
Photocatalytic water splitting. A) Schematic of water splitting using semiconductor photocatalyst. B) Band structure of semiconductors and redox potentials of water splitting. Reproduced with permission.[[qv: 13a]] Copyright 2009, RSC.

Photocatalytic water splitting has fundamental requirements for photocatalysts including their band gaps and band levels. For water reduction reaction, the conduction band needs to be at a potential less than 0 V vs. NHE (H^+^/H_2_) while the valence band needs to be at a potential more than 1.23 V that corresponds to light of 1008 nm (**Figure** **2**). As ultraviolet light has much higher photonic energy than visible light, ultraviolet‐based photocatalysts perform better per photon for hydrogen production via solar water splitting than visible light‐based ones. So far, most of the reported photocatalysts are only active under ultraviolet light irradiation. However, ultraviolet light (<400 nm) only accounts for 4% of the total solar energy whereas visible light (400–800 nm) and infrared light (>800 nm) account for 53% and 43% of the total solar energy, respectively. As ultraviolet light accounts for only a small portion of solar energy, it is critical to rationally design and fabricate photocatalysts that are not only active to harvest more visible or infrared light but also effective to improve their low solar‐to‐hydrogen conversion efficiency over a broad spectral range. It is understandable that even a less efficient photocatalyst that absorbs visible light can be more useful than a more efficient photocatalyst absorbing solely ultraviolet light.

*Design of Photocatalysts*: Typically, photocatalysts are semiconductor materials in powders or colloidal forms, which can integrate with dopants and/or cocatalysts to optimize their performance. When irradiated with photons that have energy equal to or above the band gaps of semiconductors, the photogenerated electrons and holes in the respective conduction and the valence band can cause redox reactions. In case that they are transferred to water molecules for water splitting, the electrons reduce water to form hydrogen while the holes oxidize water to form oxygen. However, it should be noted that recombination with the opposite charges can happen and this significantly lower the water splitting efficiency.^8^ In addition to the band structure of semiconductors, other bulk and surface properties that also strongly affect the recombination process of photogenerated electrons and holes are to be taken into consideration.^8^ The crystalline photocatalysts with a low number of defects are beneficial for water splitting because the defects can act as recombination sites between photogenerated electrons and holes. For example, Kong et al. demonstrated that high temperature calcination can eliminate lattice stress in TiO_2_ to reduce defects and achieve better photocatalytic efficiency.^9^ On the other hand, the crystalline photocatalysts with smaller particle size have the shorter distance for the photogenerated electrons and holes to quickly migrate to the active reaction sites on the surface as compared to those with bigger particle size, thus lowering the recombination probability before water splitting. Meanwhile, different shapes such as two dimensional nanostructures are expected to lower the recombination rate of photogenerated electron–hole pairs for giving higher photocatalytic activity.^10^ In case that the photogenerated electrons and holes possess thermodynamically sufficient potentials for water splitting, the recombination may still occur when there is lack of suitable active sites on the surface for water splitting. Therefore, the loading of cocatalysts on semiconductors is important to introduce the active sites while suppressing the charge recombination and the reverse reaction in water splitting.^11^ In addition to the cocatalysts, sacrificial agents such as various organic/inorganic electron donors also play a significant role in influencing their photocatalytic activity for water splitting reaction. The use of sacrificial agents can greatly minimize the charge carrier recombination by scavenging the photogenerated holes. Moreover, in the absence of oxygen, the back reaction to produce water is suppressed and thus hydrogen yield is improved.^12^

*Choices of Photocatalysts*: Photocatalysts are made up of different elements with different purposes to pursue the ultimate goal for improving water splitting performance. Generally, most photocatalysts including metal oxides, sulfides and nitrides for water splitting contain metal cations with d^0^ and d^10^ configurations. For examples, transition metal oxides with d° configuration mainly consist of empty d orbitals of metal cations in their conduction bands, whereas metal oxides with d^10^ configuration consists of hybridized orbitals of empty s and p orbitals of metal cations in their conduction bands. On the other hand, the valence bands of metal oxides is mainly composed of an O 2p orbital while the valence bands of metal sulfides and nitrides are usually composed of S 3p and N 2p orbitals, respectively.^13^ Alkali, alkaline earth and some lanthanide ions are also used as components in photocatalysts though they do not directly contribute to the band formation but simply construct the crystal structure (e.g. perovskite). Moreover, a number of transition metal cations with partially filled d orbitals such as Cr^3+^, Ni^2+^ and Rh^3+^ are used to dope photocatalysts so as to form some impurity levels in band gaps. In addition, some noble metals and transition metal oxides (e.g. Ru, Rh, Pd, Pt, Au, Ag, NiO) are used as cocatalysts for hydrogen evolution reaction (HER), while noble metal oxides (e.g. RuO_2_ and IrO_2_) work as excellent cocatalysts for oxygen evolution reaction (OER).[[qv: 11,13a,14]]

### 4.1 Metal Oxide Photocatalysts

**Figure 3:**
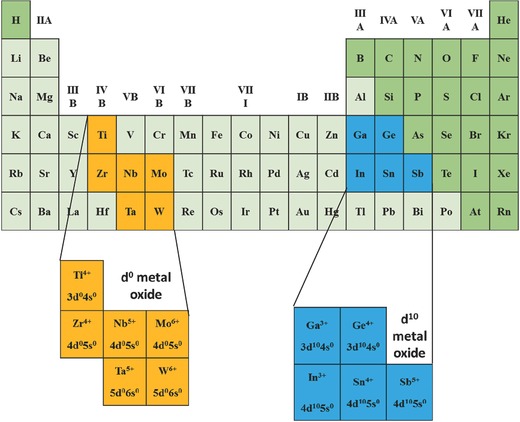
d^0^ and d^10^ transition metal photocatalyst for hydrogen production.

Various water splitting photocatalysts are mainly those transition metal oxides containing metal cations with d^0^ and d^10^ configurations. The d^0^ metal oxides are from IVB group (Ti^4+^, Zr^4+^), VB group (Nb^5+^, Ta^5+^) and VIB group (Mo^6+^, W^6+^) while the d^10^ metal oxides are from IIIA group (Ga^3+^, In^3+^), IVA group (Ge^4+^, Sn^4+^) and VA group (Sb^5+^) (**Figure** **3**).

#### 4.1.1 Group IVB Metal Oxides

Group IVB metal oxides such as TiO_2_ 15 and ZrO_2_ 16 have been widely investigated as photocatalysts for water splitting reaction. TiO_2_ is more photoactive in the form of anatase as compared to rutile probably due to higher reduction potential of photogenerated electrons resulting from the more negative conduction band of anatase than that of rutile. With a large band gap (3.0 eV for rutile and 3.2 eV for anatase), TiO_2_ can only absorb ultraviolet light under solar illumination.^17^ TiO_2_ was first employed as photocatalyst for water splitting with the assistance of an external bias. As TiO_2_ is incapable of splitting water, much work has been devoted to modify the electronic structure of TiO_2_ by modifications such as doping with other elements or loading with cocatalysts, which can achieve better photocatalytic activity in visible light region through disrupting the ordered lattice structure of TiO_2_ and building new energy states within the band gap for photoexcitation with lower energy. However, it remains challenging to develop a simple and economic strategy to synthesize excellent TiO_2_‐based photocatalysts for efficient hydrogen production under visible light irradiation. In this section, we summarize the recent progress in the fabrication of various TiO_2_‐based materials for enhancing photoconversion efficiency in visible light region through doping with anions, incorporating with cations, loading with cocatalysts, reducing/disordering processes, hybridizing with other materials, etc.

*Nonmetal‐Doped TiO_2_*: Tremendous efforts have been made to introduce nonmetal elements (i.e. B, C, N, P, S, halides) into TiO_2_ as acceptor states above the valence band for greatly improving light absorption and charge transport properties.^18^ The substitution of O with N into TiO_2_ for mixing N 2p with O 2p states, which can narrow the band gap of TiO_2_ by shifting upward the edge of valence band.[[qv: 18a]] The doping greatly improved visible light absorption with a shift of absorption onset from 380 to 600 nm.[[qv: 18b]] The doping also controlled the preferred orientation with a large percentage of exposed (211) facet, resulting in a remarkable increase in hydrogen production rate.[[qv: 18l]] With large ionic radius, it is difficult to incorporate S into TiO_2_ for inducing a similar narrowing of band gap. This is also supported by a much larger formation energy required for the substitution of S than that of N.[[qv: 18a]] It was also reported that C and P dopants introduce deep states in the band gap of TiO_2_, which suppress the transfer of photogenerated charge carriers to the surface of photocatalysts.[[qv: 18a]] The doping of halides such as F^−^, Cl^−^ and Br^−^ into TiO_2_ also increase the optical response in visible spectral region, resulting in higher photocatalytic activity for water splitting reaction.[[qv: 18d,g]] Further, different doping strategies and dopant concentrations have strong effects in enhancing the photocatalytic activity of TiO_2_.[[qv: 18e,i,k,l]] Cao et al. demonstrated that selective N doping in TiO_2_ electrode improved electronic conductivity and enhanced the incident photon‐to‐electron conversion efficiency in UV light region as compared to the uniform N doping.^18^

**Figure 4:**
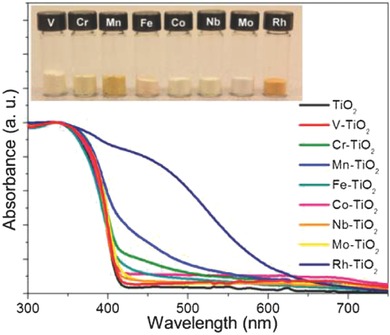
Picture and UV‐vis absorption spectra of various transition metal‐doped TiO_2_ nanowires. Reproduced with permission.[[qv: 20b]] Copyright 2013, ACS.

*Metal‐Doped TiO_2_*: Extensive efforts have been made to introduce metal ions into TiO_2_ as donor states below the conduction band for effectively enhancing the photocatalytic activity of water splitting reaction. There is an earlier report on a systematic doping of various metal ions in TiO_2_ nanoparticles.^19^ Among them, the doping with V^4+^, Fe^3+^, Mo^5+^, Ru^3+^, Rh^3+^, Re^5+^ and Os^3+^ increased the photoactivity of TiO_2_ while the doping with Al^3+^ and Co^3+^ reduced the photoactivity of TiO_2_.^19^ The photoactivity is associated with the ability of dopants to trap and transfer electrons or holes, which depends on the concentration, distribution and energy level of dopants in TiO_2_ together with their d electron configuration. In comparison, the doping with Li^+^, Mg^2+^, Al^3+^, Zn^2+^, Ga^3+^, Zr^4+^, Nb^5+^, Sn^4+^, Sb^5+^ and Ta^5+^ exhibits a little effect on photoactivity because of their closed shell electron configurations that are very stable to make electrons/holes trapping unfavorable. To date, there are many emphasized studies on the photoactivity of 3d transition metal‐doped TiO_2_ 20 because the incorporation of such dopants can extend the light absorption edge of TiO_2_ from ultraviolet to visible spectral region (**Figure** **4**).[[qv: 20b]] The doping with a series of lanthanide ions (e.g. Eu^3+^, Gd^3+^, Ho^3+^ and Yb^3+^) and the co‐doping with Nd^3+^/Er^3+^, Nd^3+^/Eu^3+^ or Eu^3+^/Ho^3+^ pairs were reported to improve the photocatalytic activity of TiO_2_.^21^ The combined use of TiO_2_ with a second oxide in heterostructures such as SnO_2_/TiO_2,_ 22 ZrO_2_/TiO_2_,^23^ Cu_x_O/TiO_2_,^24^ ZnO/TiO_2_,^25^ Ag_x_O/TiO_2_,^26^ and MTiO_3_/TiO_2_ (M = Ca, Sr, Ba) also improved the photocatalytic activity of TiO_2_ to exhibit superior photocatalytic HER.^27^

*Cocatalyst‐loaded TiO_2_*: Besides the band gap engineering to tune the light absorption of TiO_2_, the loading with cocatalysts can also enhance the photocatalytic activity of TiO_2_ for water splitting reaction. The cocatalysts play a role in extracting photogenerated charge carriers, hosting active sites for photocatalytic water reduction or oxidation reaction, suppressing photocorrosion, and thereby improving the stability of photocatalysts. Noble metals such as Ru, Rh, Pd, Ag, Pt and Au are commonly used as efficient cocatalysts for photocatalytic water reduction reaction.^11^, ^14^, ^28^ As the noble metals have lower Fermi level than that of TiO_2_, the photoexcited conduction band electrons of TiO_2_ can transfer to the metals (i.e. the metals can trap the photoexcited electrons of TiO_2_) when in contact while the photogenerated valence band holes remain on the TiO_2_. Hence, the presence of such cocatalysts greatly minimizes the possibility of electron‐hole recombination, resulting in efficient electron‐hole separation and strong photocatalytic reaction.^29^ Among these different cocatalysts, Pt is the most effective promoter for HER due to its large work function and low overpotential.[[qv: 11,14,28c]] Sreethawong et al. reported enhanced photocatalytic hydrogen production over Pt‐supported TiO_2_ while no appreciable HER was observed from pure TiO_2_.^30^ Wu et al. reported that N‐doped TiO_2_ nanofibers loaded with Pt nanoparticles exhibited high photocatalytic efficiency in hydrogen production under ultraviolet light irradiation.^31^ Zhao et al. conducted a computational investigation of Pt‐loaded anatase TiO_2_ to imply that the Pt cocatalyst is able to suppress the recombination of photogenerated electron‐hole pairs and also promotes visible light absorption due to the surface plasmon resonance occurring on the surface of Pt/TiO_2_ photocatalyst.^32^

Similarly, Au has also been widely investigated as a cocatalyst on TiO_2_ for photocatalytic hydrogen production. Murdoch et al. studied Au‐loaded TiO_2_ photocatalyst and revealed that Au nanoparticles in the size range of 3 to 30 nm were very active in hydrogen evolution while Au nanoparticles in the size range of 3 to 12 nm did not affect the hydrogen evolution rate.^33^ Rosseler et al. observed the enhancement of hydrogen evolution rate at an increased loading of Au nanoparticles on TiO_2_, which is partially due to the increase of particle size from 3 to 8 nm.^34^ Sakthivel et al. reported that the photocatalytic activity increased with the increase of noble metal loading up to an optimal level due to the decreased recombination of electrons and holes.^35^ In addition to the reduced photoabsorption of TiO_2_, the overloaded noble metals on TiO_2_ acted as recombination centers of electrons and holes, causing detrimental effect to photocatalyst performance.^35^ On the other hand, noble metal oxides such as RuO_2_ and IrO_2_ are used as efficient cocatalysts on TiO_2_ for water oxidation reaction through improving its photocatalytic activity in oxygen production. Although these noble metals or their oxides as cocatalysts exhibited excellent photocatalytic activity, they are not practical materials to be used for hydrogen production at large scale due to their high cost. Therefore, in recent years, great efforts have been made to construct other kinds of cocatalysts from cheap and earth‐abundant elements for assisting the photocatalytic water splitting.^14^

**Figure 5:**
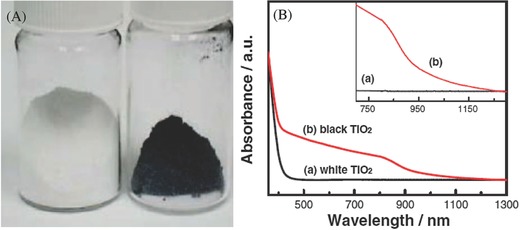
A) Unmodified white and hydrogenated black TiO_2_ nanocrystals and B) spectral absorbance of the white and black TiO_2_ nanocrystals. Reproduced with permission.[[qv: 36a]] Copyright 2011, AAAS.

*Disordered TiO_2_*: Black TiO_2_ was synthesized by hydrogenation process for greatly shifting the top edge of valence band of white TiO_2_, drastically narrowing its band gap and thus displaying strong absorption in visible and infrared regime.^36^ Naldoni et al. reported the band gap narrowing of black TiO_2_ nanoparticles (crystalline core and disordered shell) in the co‐presence of surface disorder and oxygen vacancies.[[qv: 36d]] The hydrogenated TiO_2_ exhibits excellent solar‐driven activity and stability for the photocatalytic production of hydrogen.^37^ Chen et al. engineered the surface disorder of TiO_2_ (crystalline nanocrystals as a core and disordered surface layer where dopants was introduced) for improving visible and infrared absorption (**Figure** **5**).[[qv: 36a]] In addition to the hydrogenation process, Wang et al. employed aluminum reduction to synthesize black TiO_2_ with crystalline core and amorphous shell structure to significantly improve visible and near‐infrared photoabsorption.[[qv: 36f]] The oxygen‐deficient shell was responsible for wide‐spectrum light absorption that consequently boosted the photocatalytic activity of water splitting. By combining hydrogenation and nitridation treatments, the absorption edge of TiO_2_ was shifted further to longer wavelength region and its photocatalytic activity was significantly enhanced due to the synergistic effect of co‐doping with H and N.[[qv: 36c]]

*Reduced TiO_2_*: Optical absorption of TiO_2_ from ultraviolet to visible spectral region can also be improved by introducing surface defects, which are dominated by Ti^3+^ defects together with oxygen vacancies.^38^ Self‐doping with Ti^3+^ avoids the introduction of unfavorable carrier recombination centers as well as thermal instability associated with the dopants. The role of Ti^3+^ defects in TiO_2_ shifts the top of valence band upwards for narrowing its band gap.^39^ It is demonstrated that the reduced structure such as Ti^3+^‐doped TiO_2_ can potentially lower the recombination rate of electron‐hole pairs due to the presence of Ti^3+^ and oxygen vacancies that are able to trap photoexcited electrons on the surface.[[qv: 38b,c]]

*Metal/TiO_2_ Hybrids*: With a large band gap, TiO_2_ only has strong absorption in ultraviolet spectral region, limiting its solar photocatalytic efficiency by its inability to absorb visible or infrared light. The integration of TiO_2_ with plasmonic metal nanoparticles (e.g. Au and Ag) is expected to improve its photocatalytic performance via plasmon‐enhanced light harvesting in longer wavelength with enhanced charge separation.[[qv: 28c,40]] Kowalska et al. showed that the visible light‐induced photocatalytic activity of Au/TiO_2_ hybrids was attributed to the surface plasmon excitation of Au on TiO_2_.^41^ Christopher et al. demonstrated that the photocatalytic activity of Au/TiO_2_ hybrids was strongly dependent on the size and shape of optically active Ag nanostructures. For instance, Ag nanocubes offer higher enhancement compared to Ag nanospheres and nanowires of similar size with identical Ag mass due to higher scattering efficiency.

Awazu et al. prepared plasmonic photocatalysts by depositing TiO_2_ on silica‐coated Ag nanoparticles (i.e. Ag@SiO_2_), demonstrating the enhanced photocatalytic activity of TiO_2_ under near‐ultraviolet light irradiation. The photocatalytic activity was enhanced with a decreased thickness of silica shell because the near field enhancement of Ag nanoparticles was strongest in close proximity to their surface.^42^ Kumar et al. also investigated the proximity effects of near field enhancement by fine‐tuning silica thickness to separate Ag nanoparticles from TiO_2_ thin films. Similarly, there is an increased near field enhancement with decreasing silica thickness, leading to an enhanced photocatalytic efficiency.^43^ Lee et al. investigated the photocatalytic behavior of bare and silica‐coated Au nanoparticles (i.e. Au@SiO_2_) loaded onto TiO_2_ by varying the size of Au nanoparticles (3, 7 and 17 nm) and the thickness of silica shells.^44^ Interestingly, 3‐nm Au‐loaded TiO_2_ shows the highest activity among all the bare Au‐loaded TiO_2_ whereas 17‐nm Au@SiO_2_‐loaded TiO_2_ show the highest activity among all the Au@SiO_2_‐loaded TiO_2_. In these Au/SiO_2_/TiO_2_ systems, the space charge separation is blocked by insulating silica shell and thus localized surface plasmon resonance plays a major role in enhancing photocatalytic activity.

**Figure 6:**
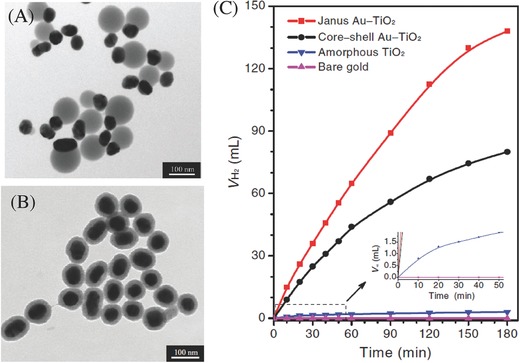
TEM images of Janus (A) and core‐shell (B) Au‐TiO_2_ nanostructures. C) Volume of hydrogen generated (V_H2_) under visible‐light irradiation from a tungsten halogen lamp using Janus and core‐shell Au‐TiO_2_ nanostructures, as well as amorphous TiO_2_ and bare gold nanoparticles (50 nm). Reproduced with permission.^45^

Seh et al. also reported the enhanced photocatalytic activity via the strong localization of plasmonic near fields in Janus Au/TiO_2_ nanostructures with uniform size of Au nanoparticles ranging from 30 to 70 nm, which were found to outperform those using 5 nm Au nanoparticles with a 4–7 fold increase in hydrogen generation under visible light irradiation.^45^ The disparity in photocatalytic activity is attributed to the stronger plasmonic near‐field enhancements and optical absorption enhancements of the larger Au nanoparticles in greater contact with TiO_2_ nanoparticles (**Figure** **6**). Cronin et al. demonstrated that the integration of Au nanoparticles with TiO_2_ films yielded up to 66 times increase in photocurrent under visible light irradiation.^46^ A reverse trend with a 4‐fold decrease under ultraviolet light irradiation was observed instead via the integration of Au nanoparticles. By simulating electromagnetic field enhancements, the increase in photocatalytic activity was attributed to the enhanced local electric fields near the TiO_2_ surface as opposed to direct charge transfer.

*Ti‐Based Perovskite*: With a cubic perovskite‐type structure, SrTiO_3_ has been widely used to split water under ultraviolet light illumination without external bias due to its high conduction level. The redox potentials of the photogenerated electrons and holes are powerful enough to facilitate the production of hydrogen and oxygen.^47^ Different dopants have been introduced as cocatalysts for SrTiO_3_ photocatalysts to effectively enhance the water splitting reaction.^48^ Puangpetch et al. designed various SrTiO_3_–based photocatalysts loaded with different metal cocatalysts (e.g. Fe, Ni, Ag, Pt, Au, Ce) for photocatalytic hydrogen production under both ultraviolet and visible light irradiation.[[qv: 48a,b]] Among these metal cocatalysts, Ni, Ag, Pt and Au showed positive effect on the photocatalytic activity.[[qv: 48b]] Au was found to be the best cocatalyst (1 wt% Au exhibited the highest photocatalytic activity for hydrogen production) due to its electrochemical properties compatible with SrTiO_3_–based photocatalysts together with its visible light harvesting enhancement. Also, NiO_x_[[qv: 48c]] and Rh^49^ are suitable cocatalysts for SiTiO_3_ photocatalyst. In particular, NiO_x_ did not cause the backward water splitting reaction between H_2_ and O_2_ to form water, being different from Pt.[[qv: 13a]] In addition, the doping of C, N and S into SiTiO_3_ exhibited effective enhancement in visible light absorption.^50^ The co‐doping of C and S into SrTiO_3_ was reported to shift the absorption edge from 400 to 700 nm, exhibiting higher photocatalytic activity than pure SrTiO_3_.[[qv: 50c]]

With a cubic perovskite‐type structure, La_2_Ti_2_O_7_ has also been widely studied in water splitting reaction under ultraviolet light irradiation due to its wide band gap of 3.8 eV.^51^ To enhance visible light absorption, metal elements are used to dope into La_2_Ti_2_O_7_ by tuning the band gap levels to significantly narrow its band gap. Hwang et al. reported that transition metal‐doped La_2_Ti_2_O_7_ such as Cr or Fe has visible light absorption because their 3d states appeared in the band gap.^52^ Similarly, Kim et al. reported that alkaline earth metal‐doped La_2_Ti_2_O_7_ such as Ba, Sr or Ca remarkably enhanced the photocatalytic activity for water splitting reaction.^53^ Liu et al. investigated mono‐ and co‐doping with cationic (V, Nb, Ta) and anionic (N) elements into La_2_Ti_2_O_7_ for tuning electronic structure through hybrid density functional study.^54^ It was found that mono‐doping created impurity states in the band gap that promoted the photogenerated electron‐hole recombination. However, the cationic–anionic mediated co‐doping could remove such impurity states by charge compensation, and this is promising for visible light photocatalysis. It was suggested that the water oxidation and reduction reactions were thermodynamically favorable for the anionic‐cationic [(N, Nb) and (N, Ta)] co‐doped La_2_Ti_2_O_7_ systems. On the other hand, with a higher p orbital energy than that of oxygen, nonmetal elements were used to dope into La_2_Ti_2_O_7_ for improving visible light photocatalysis. Liu et al. reported the anionic‐anionic mediated co‐doping in La_2_Ti_2_O_7_ for visible light photocatalysis, which lowers its band gap much more as compared to the anionic mono‐doping. Moreover, the calculated defect formation energy showed that the co‐doped systems were more stable than their respective mono‐doped systems.^55^

#### 4.1.2 Group VB Metal Oxides

As two important metal oxides of Group VB, Ta_2_O_5_ is weakly active but Nb_2_O_5_ is not active at all for water splitting reaction under ultraviolet light irradiation. Their modifications with cocatalysts (e.g. Pt, Au, NiO, RuO_2_) are required to greatly stimulate the water splitting reaction. On the other hand, metal niobates and tantalates (alkaline, alkaline earth and transition metals) are demonstrated to exhibit photocatalytic activity for water splitting. Also, vanadium dioxide and mixed metal vanadates were reported as water splitting photocatalysts for hydrogen generation.^56^

*Cocatalyst‐Loaded Nb_2_O_5_ and Ta_2_O_5_*: With a wide band gap of ≈3.4 eV, Nb_2_O_5_ only becomes active for water splitting under ultraviolet light irradiation when loaded with metals or metal oxides as cocatalysts, which effectively delay electron and hole recombination rate for enhancing hydrogen production efficiency.^57^ Lin et al. studied a series of cocatalysts such as Pt, Au, Cu and NiO nanoparticles loaded on mesoporous Nb_2_O_5_ for hydrogen evolution in an aqueous methanol solution under ultraviolet light irradiation.[[qv: 57b]] The photocatalytic activity of Pt‐loaded Nb_2_O_5_ exhibited the highest hydrogen production efficiency, which was 2.2, 2.9 and 6.5 times as much as that when loaded with Au, Cu and NiO cocatalysts, respectively. In addition, CuO was also used as an effective cocatalyst in porous Nb_2_O_5_ for hydrogen production.[[qv: 57c]] In comparison, Ta_2_O_5_ alone can only produce a very small amount of hydrogen from water splitting under the band gap irradiation of 4.0 eV. Cocatalyst such as NiO and RuO_2_ are required to improve the photocatalytic activity for water decomposition. For example, mesoporous Ta_2_O_5_ loaded with NiO was an active catalyst for photocatalytic water decomposition.^58^

*Metal Niobates*: Alkaline metals are used to prepare alkaline niobates including lithium, sodium, potassium and rubidium niobates as photocatalysts for water splitting under ultraviolet and visible light irradiation.^59^ Saito et al. reported higher hydrogen evolution activity of lithium niobate (LiNbO_3_) nanowires than that of bulky counterpart, which were required to be milled for increasing surface area but this process also caused mechanical destruction which resulted in a decrease in photocatalytic activity.[[qv: 59d]] Li et al. reported the increased photocatalytic activity of NaNbO_3_ corresponding with a decrease in particle size due to shorter diffusion length of photogenerated electrons.[[qv: 59b]] Also, the rectangular prisms and cubic particles have higher photocatalytic activity than that of spherical particles because of a larger number of edges and corners on their surface that usually worked as active sites for catalytic reactions. Domen et al. demonstrated the first example of alkaline niobates, potassium niobate (K_4_Nb_6_O_17_) as a photocatalyst to achieve high activity of water splitting in an aqueous methanol solution without any assistance from cocatalyst such as noble metals.[[qv: 59a]] The loading with various metals such as Ni,^60^ Au,^61^ Pt^62^ and Cs^63^ as cocatalysts effectively improved the photocatalytic activity of K_4_Nb_6_O_17_ for HER. Further, rubidium niobate (Rb_4_Nb_6_O_17_) was also reported to exhibit a high photocatalytic activity for water splitting.^64^

Alkaline earth metals are also used to prepare alkaline earth niobates including calcium, strontium and barium niobates as photocatalysts for water splitting under ultraviolet and visible light irradiation.^65^ The alkaline earth niobates such as Ca_2_Nb_2_O_7_,^66^ Sr_2_Nb_2_O_7_,^66^, ^67^ Sr_5_Nb_4_O_15_ 68 and Ba_5_Nb_4_O_15_ 68 exhibited high photocatalytic activities in water splitting under ultraviolet light irradiation. Particularly, Ca_2_Nb_2_O_7_ and Sr_2_Nb_2_O_7_ with highly donor‐doped (110) layered perovskite structures demonstrated high quantum yields of 7% (<288 nm) and 23% (<300 nm), respectively.^66^ Chen et al. synthesized Sr_2_Nb_2_O_7_ nanoribbons and Sr_2_Nb_2_O_6_ nanorods as efficient water splitting photocatalysts under ultraviolet light irradiation.[[qv: 67b]] Pt‐loaded Sr_2_Nb_2_O_7_ nanoribbons and Sr_2_Nb_2_O_6_ nanorods with large Brunauer‐Emmett‐Teller surface areas exhibited high quantum yields of 32% and 19%, respectively. Further, NiO‐loaded Ba_5_Nb_4_O_15_ plate‐like nanostructures with a layered perovskite structure were reported to exhibit a high quantum yield of 17% at 270 nm for water splitting.^68^ Recently, Rh‐doped calcium niobate nanosheets were prepared by exfoliating layered KCa_2_Nb_3–x_Rh_x_O_10−δ_ and demonstrated high photocatalytic activity for hydrogen generation in an aqueous methanol solution without loading with a cocatalyst. The hydrogen production rate for the Rh‐doped nanosheets was 165 times more than that of the parent Rh‐doped layered oxide. The quantum efficiency at 300 nm was as high as 65%.^69^

Meanwhile, transition metals are used to prepare transition metal niobates as photocatalysts for water splitting. Kudo et al. synthesized ZnNb_2_O_6_ consisting of d^10^ and d^0^ metal ions, exhibiting negligible photocatalytic activity under ultraviolet light irradiation. NiO‐loaded ZnNb_2_O_6_ showed high photocatalytic activity after pretreatment with hydrogen reduction and subsequent oxygen oxidation at appropriate temperatures.^70^ Other niobates photocatalyst such as Bi_3_NbO_7_ were demonstrated to possess photocatalytic activity to evolve hydrogen from water under visible light.^71^ Furthermore, mixed metal niobates were also synthesized for photocatalytic water splitting. Chen et al. demonstrated the photocatalytic activity of ABi_2_Nb_2_O_9_ (A = Ca^2+^, Sr^2+^, Ba^2+^)[[qv: 65a]] under ultraviolet light irradiation for both HER and OER in aqueous solutions containing sacrificial reagents (methanol and Ag^+^). The photocatalytic activities decreased in the order of SrBi_2_Nb_2_O_9_ > BaBi_2_Nb_2_O_9_ > CaBi_2_Nb_2_O_9_. Other mixed metal niobates including A_2_BiNbO_7_ (A = In^3+^, Ga^3+^)^72^ and ABi_2_NbO_7_ (A = Al^3+^, Ga^3+^, In^3+^, Y^3+^, Ce^3+^, Gd^3+^, Sm^3+^, Nd^3+^, Pr^3+^, La^3+^)^73^ were reported to split water into hydrogen and oxygen under ultraviolet light irradiation. In particular, the rare earth‐incorporated Bi_2_NbO_7_ exhibited a decreased trend in the photocatalytic activity with increasing their ionic radius because smaller ionic radius of rare earth led to the formation of a narrower band gap that facilitated easier excitation for an electron from valence band to conduction band.[[qv: 73b]] Moreover, BaNi_1/3_Nb_2/3_O_3_ and BaZn_1/3_Nb_2/3_O_3_ were used to split water under ultraviolet light irradiation^74^ while BaIn_1/3_Nb_2/3_O_3_ and BaCo_1/3_Nb_2/3_O_3_ were employed to split water under visible light irradiation.[[qv: 65b,c]]

*Metal Tantalates*: Kato et al. reported high photocatalytic activities of alkaline tantalates, ATaO_3_ (A = Li^+^, Na^+^, K^+^) for water splitting under ultraviolet light irradiation.^75^ The modification with cocatalysts such as NiO[[qv: 75c]] and Au^61^ enhanced the photocatalytic activities for water splitting. Among them, NiO‐loaded NaTaO_3_ showed the most efficient water splitting in pure water.^76^ The photocatalytic activity of the NiO‐loaded NaTaO_3_ was drastically increased by 9 times after further doping with La, yielding the highest quantum yield (56% at 270 nm) for water splitting under ultraviolet light irradiation without the use of sacrificial reagents.^76^ This is because the La‐doping created the ordered surface nanostructures with characteristic steps that suppressed the recombination of the photoexcited electron‐hole pairs. Moreover, the partial substitution of Ta in NiO‐loaded KTaO_3_ with Ti^4+^, Zr^4+^ or Hf^4+^ in group IVB effectively improved the photoactivity through controlling the charge density in KTaO_3_.^77^ In particular, NiO‐loaded KTaO_3_ doped with 8 mol% Zr^4+^ exhibited higher photocatalytic activity than the well‐known Pt/TiO_2_ photocatalyst reported in literature.

Kato et al. reported the high photocatalytic activities of alkaline earth tantalates ATa_2_O_6_ (A = Mg^2+^, Ba^2+^) for water splitting under ultraviolet light irradiation.[[qv: 75a]] Liang et al. demonstrated that strontium tantalates such as Sr_0.25_H_1.5_Ta_2_O_6_.H_2_O exhibited much higher photocatalytic activity than strontium niobates due to the high reduction ability of photogenerated electron and high electron mobility in conduction band.^78^ Recently, barium tantalate composite consisting Ba_5_Ta_4_O_15_/Ba_3_Ta_5_O_15_/BaTa_2_O_6_ were found to be active photocatalysts for water splitting in the absence of cocatalyst under ultraviolet light irradiation.^79^ The addition of core‐shell Rh/Cr_2_O_3_ cocatalysts further enhanced the photocatalytic activity of the photocatalyst for pure water splitting, achieving up to 70% higher than that of Ba_5_Ta_4_O_15_ itself. It was suggested that the enhanced activity for water splitting was induced by the combination of effective charge carrier separation and improved electron transfer in the highly crystalline barium tantalate composite modified with core‐shell Rh/Cr_2_O_3_ cocatalyst.

Furthermore, mixed metal tantalates such as MLnTa_2_O_7_ (M = H^+^, Na^+^, Rb^+^, Cs^+^; Ln = La^3+^, Pr^3+^, Nd^3+^, Sm^3+^) were reported to demonstrate their photocatalytic activity for water splitting.^80^ Under ultraviolet light irradiation, RbNdTa_2_O_7_ with partially occupied 4f shell was first reported as an active photocatalyst for stoichiometric H_2_/O_2_ evolution from pure water without the use of metal cocatalyst loading and sacrificial agents.[[qv: 80a]] It was observed that the sequence of photocatalytic activity followed Nd > Sm >> La ≈ Pr, suggesting that partially occupied lanthanide 4f shell played an important role in photocatalytic reaction. This is attributed to the shift of 4f levels from conduction band edge to covalent band edge with increasing the number of 4f electrons, greatly influencing the band gap energy.[[qv: 80d]] Other lanthanide‐doped tantalates such as La_1/3_TaO_3_ with NiO cocatalyst were also reported to evolve hydrogen under ultraviolet light irradiation from water.^81^

#### 4.1.3 Group VIB Metal Oxides

Group VIB metal oxides, in particular, W and Mo‐based ones were found to be active for water splitting under ultraviolet light irradiation though they also have optical absorption in visible region. It was interesting to study tetrahedral WO_4_ as an active site for water splitting because most of conventional transition metal oxides were composed of octahedral structure. Photocatalytic activity of PbWO_4_ for water splitting was achieved with a combination of crystallized PbWO_4_ with a high dispersion of RuO_2_ particles.^82^ The findings also suggested the importance of hybridized d^10^S^2^ configuration of Pb^2+^ with d° configuration of W^6+^ in water splitting because no significant activity was observed for CaWO_4_ and ZnWO_4_. PbMoO_4_ was also photoactive for sacrificial water splitting with the assistance of surface‐deposited platinum.^83^ Moreover, metal tungstates (e.g. Na_2_W_4_O_13_,^84^ Bi_2_W_2_O_9_,^85^ Bi_2_WO_6_,^85^ ZrW_2_O_8_ 86) and metal molybdates (e.g. (AgBi)_0.5_MoO_4_,^87^ (NaBi)_0.5_MoO_4_ 87) showed photocatalytic activity for water splitting under ultraviolet light irradiation.

#### 4.1.4 Group IIIA, IVA and VA Metal Oxides

Group IIIA metal oxides containing metal ions (e.g. Ga^3+^, In^3+^) with d^10^ configuration and relatively wide band gap were active for water splitting under ultraviolet light irradiation. Ga_2_O_3_ has five different polymorph phases (α, β, γ, δ, ε). Among them, β‐Ga_2_O_3_ is the most thermodynamically stable.^88^ The photocatalytic activity of β‐Ga_2_O_3_ supported with Ni cocatalyst was effectively improved by the addition of a small quantity of metal cations such as Ca, Sr, Ba, Cr, Ta and Zn ions.^89^ Particularly, the addition of Zn ions enhanced the photocatalytic activity most remarkably. The photocatalytic activity of bare Ga_2_O_3_ was not influenced by the incorporation of Mg, Ni and La ions while the photocatalytic activity was negatively impacted by the incorporation of Ti, Fe, Co, Cu, Nb and Rh ions. When loaded with Cr_2_O_3_ and CuO_x_ as cocatalysts, the photocatalytic activity of β‐Ga_2_O_3_ was greatly improved in hydrogen evolution rate.^90^ It was reported that Ga_2_O_3_ with tunable α–β phase junction significantly enhanced the photocatalytic activity over α or β phase structures due to efficient charge separation and transfer across the α–β phase junction.^91^ On the contrary, the disordered structure at the interface of γ‐Ga_2_O_3_ and β‐Ga_2_O_3_ served as defects and charge recombination centers to compromise the photocatalytic activity.^92^

Besides, Ga_2_O_3_ and In_2_O_3_ were mixed to yield mixed metal oxide Ga_1.14_In_0.86_O_3_, which displayed the high photocatalytic activity for HER in aqueous solution of methanol and OER in aqueous solution of silver nitrate.^93^ In_2_O_3_ and Y_2_O_3_ were also mixed to yield Y_1.3_In_0.7_O_3_, which exhibited high photocatalytic activity when loaded with RuO_2_ cocatalyst. The enhancement was attributed to the deformation of InO_6_/YO_6_ octahedral units as well as the lifting of conduction band levels.^94^ Other mixed metal oxides such as CaIn_2_O_4_, SrIn_2_O_4_, BaIn_2_O_4_, Sr_2_SnO_4_ and NaSbO_3_ loaded with RuO_2_ cocatalyst were studied with respect to their photocatalytic activity in HER under ultraviolet light irradiation. Among them, RuO_2_‐loaded CaIn_2_O_4_ exhibited the highest photocatalytic activity for hydrogen and oxygen evolutions from pure water.^95^ In contrast, RuO_2_‐loaded alkaline metal indates such as LiInO_2_ and NaInO_2_ were also synthesized. RuO_2_‐loaded LiInO_2_ showed poor photocatalytic activity while Ru‐loaded NaInO_2_ exhibited the photocatalytic ability to decompose water towards hydrogen generation.^96^ Groups IVA and VA metal oxides (e.g. Ge^4+^, Sb^5+^) were applied as photocatalysts for water splitting under ultraviolet light irradiation. The mixed oxides Zn_2_GeO_4_ and LiInGeO_4_ loaded with RuO_2_ cocatalyst exhibited the photocatalytic activity correlated with its dipole moments.^97^ Likewise, RuO_2_‐loaded antimonites such as NaSbO_3_, CaSb_2_O_6_, Ca_2_Sb_2_O_7_ and Sr_2_Sb_2_O_7_ were photocatalytically active towards hydrogen generation.^98^

### 4.2 Metal Chalcogenide Photocatalysts

Metal chalcogenides are attractive visible light‐sensitive photocatalysts for hydrogen production due to the relatively high conduction band position. Generally, most of the metal chalcogenide photocatalysts consist of metal cations with d^10^ configuration (e.g. group IB: Cu, Ag; group IIB: Zn, Cd; group IIIA: Ga, In; group IVA: Ge, Sn). In addition to binary metal chalcogenides, multinary metal chalcogenides have also been investigated recently.

#### 4.2.1 Group IIB Metal Chalcogenides

Group IIB metal chalcogenides including ZnS, CdS and CdSe are the most popular photocatalysts. However, the metal chalcogenides are prone to photocorrosion in aqueous solutions under irradiation. Thus numerous efforts have been made to overcome this limitation by using suitable sacrificial agents. In general, Na_2_S and Na_2_SO_3_ mixture has been widely used as sacrificial agents.

**Figure 7:**
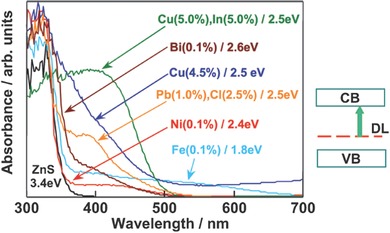
UV‐vis absorption spectrum of metal ions doped ZnS photocatalyst. Reproduced with permission.[[qv: 13a]] Copyright 2009, RSC.

*ZnS*: With a wide band gap (≈3.6 eV), ZnS can directly absorb ultraviolet light to demonstrate high photocatalytic activity in hydrogen production without the need to deposit expensive charge transfer cocatalyst like Pt or RuO_2_.^99^ A number of attempts have been made to realize visible light response of ZnS by doping with foreign elements such as Cu, Ni or Pb for exhibiting high photocatalytic activity in HER in the presence of hole scavenger even without noble metal cocatalyst.^100^ As shown in **Figure** **7**, metal ions were doped to significantly improve the visible light absorption due to the transitions of dopant levels to the conduction band of ZnS.[[qv: 13a,100a‐c]] Metal cation‐doped ZnS showed the photocatalytic activity for HER without a loading with cocatalysts such as Pt, indicating that the high conduction band of ZnS is maintained after the doping with metal cations. Besides the doping with metal ions, Muruganandham et al. first reported the co‐doping with N and C in hierarchical porous microspheres of ZnS as a visible light‐responsive photocatalyst.[[qv: 100d]]

*CdS*: With a suitable band gap (≈2.4 eV), CdS is one of the most studied metal chalcogenide photocatalysts for water splitting under visible light irradiation. The photocatalytic activity of CdS is not so effective because photogenerated electrons and holes cannot be efficiently separated and transferred.^101^ To improve the photocatalytic activity for hydrogen production, extensive researches have been focused on the preparation of CdS with different morphologies/structures, the modifications with cocatalyst, and the hybridizations with other semiconductors (to be discussed in the next section).^102^

CdS photocatalysts were prepared with various morphologies such as nanoparticles, nanospheres, nanorods, nanowires and nanosheets to improve photocatalytic hydrogen production.[[qv: 101b,103]] Sathish et al. demonstrated the size effect of CdS nanoparticles of less than 10 nm in size to alter the photocatalytic activity for hydrogen production. The smaller sized nanoparticles decrease the migration distance of the photogenerated electrons and holes to the reaction sites on the surface, which greatly reduces the recombination probability and thus increase photocatalytic activity.[[qv: 103a,b]] Li et al. reported the preparation of CdS nanospheres (solid or hollow) and nanorods in larger size for HER under visible light irradiation. As compared to hollow nanospheres and nanorods, solid nanospheres are beneficial to suppress the recombination of electrons and holes, which can quickly migrate to the reaction sites on surface to react with water and sacrificial agents for improving photocatalytic activity.[[qv: 103e]] Jiang et al. demonstrated that CdS nanowires with higher crystallinity showed higher rate of photocatalytic hydrogen production under visible light irradiation.[[qv: 103c]] Xu et al. reported ultrathin CdS nanosheets stabilized by L‐cysteine were efficient visible light‐driven photocatalyst for hydrogen production.[[qv: 101b]]

CdS photocatalysts were prepared with different structures such as crystal structure, crystallinity and defects to improve photocatalytic hydrogen production. In an early example, Matsumura et al. reported the photocatalytic activity of CdS powder with different crystal structures and found that the Pt‐loaded CdS powder with a hexagonal crystal structure was much more efficient than that with a cubic crystal structure.^104^ Bao et al. studied the photocatalytic activity of phase‐controlled CdS nanocrystals and demonstrated the highest photocatalytic activity of hexagonal CdS with good crystallinity among different phases of CdS.^105^ Jang et al. revealed that CdS nanowires with higher crystallinity exhibited higher photocatalytic efficiency because of less defects which minimize the recombination center of photoinduced electron‐hole pairs.[[qv: 103c]] Fan et al. demonstrated that high temperature calcination of hexagonal CdS in Ar atmosphere eliminated the trap energy levels for improving the photocatalytic activity, thereby decreasing the recombination rate of photogenerated charge carriers. The treated CdS exhibited 55.8 times higher in photocatalytic activity for hydrogen production under visible light irradiation.^106^

Besides the control in morphologies and structures, CdS photocatalysts were modified with cocatalysts (e.g. noble metals or noble metal compounds) to stimulate charge transfer and reduce the recombination rate of photoinduced charges for enhancing photocatalytic hydrogen production. Sathish et al. investigated various noble metal (Pt, Pd, Ru or Rh)‐loaded CdS nanoparticles on their photocatalytic hydrogen evolution rates. Pt is found to be a favorable cocatalyst for hydrogen evolution. It was observed that the hydrogen production activity of Ru‐loaded CdS nanoparticles is lower than that of the naked CdS due to the strong ruthenium‐hydrogen bond which inhibits HER on the surface of ruthenium.[[qv: 103a,b]] Bao et al. designed and prepared nanoporous CdS nanostructures (i.e. nanosheets and hollow nanorods with large surface area) loaded with Pt nanocrystals as cocatalyst. The high hydrogen production yield of ≈4.1 mmol h^–1^ under visible light irradiation (λ ≥ 420 nm) was achieved, corresponding to the apparent quantum yield of ≈60.34% measured at 420 nm. The Pt cocatalyst is significantly crucial for efficient charge separation, fast transport of photogenerated carriers, and fast photochemical reaction at the interface of CdS/electrolyte.[[qv: 103f]] Xu et al. reported that ultrathin CdS nanosheets loaded with 1 wt% PdS as a cocatalyst enhanced the apparent quantum efficiency from 1.38 to 9.62%.[[qv: 101b]]

The simultaneous loading of suitable dual cocatalysts may increase the photocatalytic activity of semiconductors. Yan et al. reported that the PdS and Pt co‐loaded CdS achieved extremely high apparent quantum efficiency of 93% at 420 nm for photocatalytic hydrogen production in the presence of sacrificial reagents. The co‐existence of PdS acting as an oxidation cocatalyst and Pt (or Pd) acting as a reduction cocatalyst is supposed to be beneficial for the efficient separation and transfer of photoexcited electrons and holes, thus contributing to the extremely high quantum efficiency. The PdS can also protect CdS from photocorrosion, and make the photocatalyst very stable under the photocatalytic reaction conditions. This co‐loading strategy of suitable dual cocatalysts demonstrates the possibility of realizing visible‐light‐responsive photocatalytic hydrogen production with a quantum efficiency approaching to the level of natural photosynthesis (95%).^107^ The loading of noble metals as cocatalysts may improve the photocatalytic hydrogen production efficiency but the high cost may limit their practical application. It is therefore necessary to explore new non‐noble metals as cocatalysts,^14^ which make the renewable hydrogen production more economical. Luo et al. prepared Ni‐doped CdS hollow spheres with enhanced photocatalytic activity and durability.^108^ Zhang et al. reported the synthesis and evaluation of low cost NiS‐loaded CdS photocatalysts under visible light irradiation. In the absence of noble metals, a high quantum efficiency of 51.3% was measured at 420 nm.^109^

**Figure 8:**
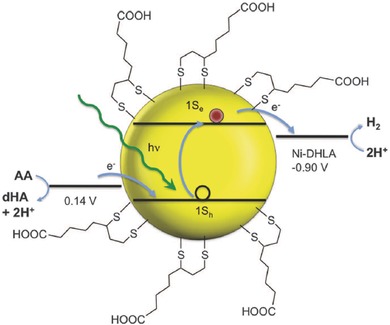
Schematic illustration of the CdSe nanocrystals capped with dihydrolipoic acid (DHLA) as the light absorber and relevant energies for H_2_ production. dHA indicates dehydroascorbic acid. Potentials are shown versus that of an NHE at pH = 4.5. Reproduced with permission.^113^ Copyright 2012, AAAS.

*CdSe*: With a suitable size‐dependent band gap, CdSe is also one of the most studied metal chalcogenide photocatalysts for water splitting under visible light irradiation. CdSe nanocrystals with size below 10 nm are also known as quantum dots, exhibiting unique quantum size effect (size‐dependent band gaps). Holmes et al. studied the relationship between the degree of quantum size confinement in suspended CdSe nanocrystals and their photocatalytic activity of water splitting.^110^ Higher hydrogen production rates were observed in CdSe nanocrystals with diameter of 2.25–3 nm. The results emphasized the dependency of charge transfer kinetics on thermodynamic driving force of the reaction, as predicted by theory, and the possibility of fine‐tuning photocatalytic activity through particle sizing. Huang et al. reported the core‐shell CdSe/ZnS quantum dots functionalized with cobaloxime for efficient hydrogen production under visible light irradiation in the presence of a proton source and a sacrificial electron donor.^111^ It was demonstrated that the quantum dots have the ability to store and donate multiple electrons to the adsorbed cobaloxime catalysts, playing a key role in improving the photocatalytic efficiency. Tongying et al. reported that the core‐shell CdSe/CdS nanowires exhibited very high photocatalytic efficiency for hydrogen generation, which is thirty times higher than that of the bare CdSe nanowires.^112^ CdSe is the active species responsible for chemical reduction processes despite of the presence of a CdS shell on it. This stems from ultrafast charge transfer between the shell and the core in CdS/CdSe nanowires. In a recent work, Han et al. demonstrated a robust and highly active CdSe nanocrystals loaded with nickel as photocatalyst for solar hydrogen generation in water. A high efficiency of >36% at the excitonic peak (520 nm) was observed with the assistance of sacrificial reagents.^113^ (**Figure** **8**)

#### 4.2.2 Multinary Metal Chalcogenides

**Figure 9:**
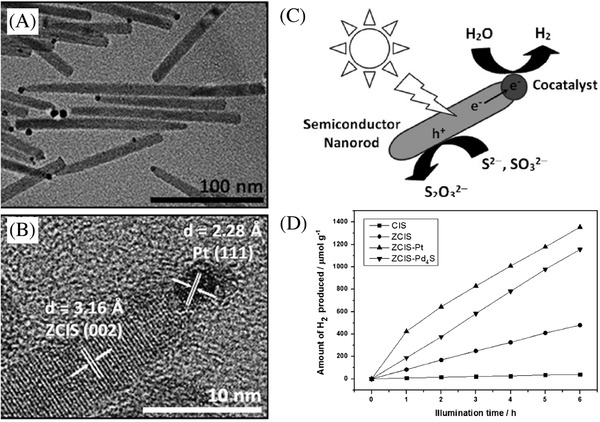
A) TEM and B) HRTEM images of ZnS‐CuInS_2_ alloy nanorods. C) Schematic depiction of photocatalytic H_2_ production from water with a photocatalyst system based on a hybrid nanostructure that consists of a semiconductor nanorod and a metallic/conducting cocatalyst. D) Photocatalytic hydrogen production under visible‐light illumination by CuInS_2_ (CIS) nanorods, ZnS–CuInS_2_ (ZCIS) nanorods, ZCIS–Pt hybrid nanocrystals, and ZCIS–Pd_4_S hybrid nanocrystals from an aqueous solution containing 0.25 m Na_2_SO_3_ and 0.35 m Na_2_S. Reproduced with permission.[[qv: 117h]]

In addition to the widely studied Group IIB metal chalcogenides, there are more multinary metal chalcogenides prepared with the combined use of two or more groups from group IB (Cu, Ag),^114^ group IIB (Zn, Cd),^115^ group IIIA (Ga, In)^116^ and group IVA (Ge, Sn).[[qv: 12c]] For example, ZnS was alloyed with other metal (e.g. Cu, Ag, In) sulfide to produce solid solutions such as ZnS–CuInS_2_,[[qv: 116b,117]] ZnS–CuS,^118^ AgInZn_7_S_9_,^119^ (AgIn)_x_Zn_2(1–x)_S_2_,^120^ (CuIn)_x_Zn_2(1–x)_S_2_,[[qv: 117a]] ZnS–CuInS_2_–AgInS_2_,^114^ and ZnS–In_2_S_3_–CuS^121^ for improving the photocatalytic properties of ZnS in the visible light.^121^ Ye et al. reported the synergistic effect of ZnS and CuInS_2_ alloy nanorods in visible light driven photocatalytic hydrogen production.[[qv: 117h]] Through alloying ZnS with CuInS_2_, it alleviated the limitation of wide band gap ZnS for visible‐light utilization and meanwhile improved the conduction band of CuInS_2_ for photocatalytic reduction of water to hydrogen. This strategy effectively modified the band structure of the semiconductors and improved the photocatalytic activity under visible light irradiation. Further, the photocatalytic activity of ZnS‐CuInS_2_ was enhanced with the loading of cocatalyst such as Pt and Pd_4_S onto ZnS‐CuInS_2_ nanorods (**Figure** **9**).

Likewise, a series of (CuIn)_x_Zn_2(1–x)_S_2_ microspheres was also reported to exhibit photocatalytic hydrogen evolution activity under visible light irradiation.^122^ The change in composition greatly modified the band gap of the solid solution. The highest photocatalytic activity was obtained from Ru‐loaded Zn_1.6_Cu_0.2_In_0.2_S_2_ with an apparent quantum yield of 15.45% at 420 nm. Besides the change in the energy band structure, surface area and crystallite size also contributed to the difference in photocatalytic performance. The alloying of CuInS_2_ with CuGaS_2_ also led to an enhancement towards photocatalytic performance because the CuInS_2_ band gap structure alone is not appropriate to overcome the reaction overpotential.[[qv: 116b]] The incorporation of Ga into CuInS_2_ effectively modified the band structure by raising the conduction band and thus providing a larger driving force to photogenerate carriers for activating the water splitting reduction reaction. However, when the concentration of Ga was too high, the band gap became wider and this reduced the concentration of photogenerated carriers available for water splitting, thus resulting in a detrimental decrease in hydrogen conversion rate. Therefore, the optimum concentration of Ga in CuIn_0.3_Ga_0.7_S_2_ was obtained to achieve the highest hydrogen generation rate. In a similar study, In was substituted with Ga in the AgGaS_2_ to modify the band gap of AgGa_1–x_In_x_S_2_.[[qv: 116a]] The highest hydrogen evolution rate was observed in AgIn_0.1_Ga_0.9_S_2_ under visible light irradiation. Also, it was suggested that the unique hierarchical microarchitectures introduced large surface area and active site, which were beneficial for the photocatalytic hydrogen evolution.

## 5 Electrocatalytic Water Splitting

The electrolysis of water has been used commercially to produce hydrogen since early 1900s, accounting for ≈4% of today's hydrogen production.^123^ This water splitting process is technologically simple but still lacks of the significant commercial impact due to high energy consumption. To improve the reaction kinetics and efficiency of water electrolysis, electrocatalysts are applied to the anode and cathode for catalyzing the electrocatalytic water reduction and oxidation reactions, respectively. Currently, noble metals are commonly employed as water splitting electrocatalysts. Ir^124^ and Ru^124^, ^125^ as well as their oxides^126^ are applied at the anode to enhance OER for water oxidation reaction while Pt is the well‐known hydrogen evolution catalyst at the cathode to enhance HER for water reduction reaction. In the recent years, great efforts have also been made to develop binary or ternary non‐noble metals or oxides in water oxidation electrocatalysts (e.g. Fe,^127^ Ni‐Fe,^128^ Ni‐Co,^129^ Ni‐Fe‐Co^130^ and CaMn_4_O_x_ 131) and non‐noble metal oxides, sulfides and phosphides water reduction electrocatalysts (MoO_3–x_,^132^ WO_2_,^133^ WO_3_,^134^ MoS_2,_ 135 WS_2_,[[qv: 135b]] CoP,^136^ Co_2_P^137^ and Ni_2_P[[qv: 137a,138]]) for cost‐competitive electrocatalysis. The non‐noble metal electrocatalysts were also extended to bifunctional types such as TiN@Ni_3_N,^139^ Ni_3_Se_2_/Ni,^140^ CoO/CoSe_2_ 141 and CoMnO@CN^142^ for both HER and OER in overall water splitting.

**Figure 10:**
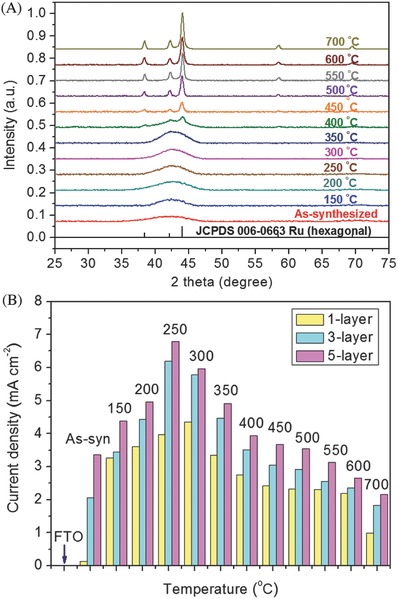
A) XRD pattern of Ru nanoparticles before and after annealing at temperatures from 150 to 700°C under argon atmosphere. Black vertical lines represent the hexagonal crystal phase of Ru (JCPDS file 06–0663). B) Electrocatalytic properties of the as‐synthesized Ru nanoparticles in their spin‐coated films (1 layer, 3 layers and 5 layers) at room temperature and their treated films under argon atmosphere at different temperatures including 150, 200, 250, 300, 350, 400, 450, 500, 550, 600, 650 and 700 °C. Linear sweep voltammetry experiments recorded at fluorine‐doped tin oxide glass substrate in sodium sulphate solution (0.1 M, pH 6) at a scan rate of 0.05 Vs^–1^. The current densities of the films were compared at 1.23 V (vs. Ag/AgCl). Reproduced with permission.^125^ Copyright 2015, IOPscience.

*Oxygen Evolution Reaction (OER)*: The integration of oxides with other metal substrates has been proven to enhance electrocatalytic water oxidation reaction. Yeo et al. examined this by depositing Co_3_O_4_ on Au as well as other metallic substrates. It was found that the OER activity of Au‐supported Co_3_O_4_ was 40 times higher than its bulk counterpart, whereas Co_3_O_4_ with other metal substrates exhibited less significant enhancement.^143^ The OER activity of Co_3_O_4_ increased with the increasing electronegativity of metal substrates in a sequence of Au > Pt > Pd > Cu > Co. The more electronegative Au promoted the oxidation of cobalt oxide to obtain an increase population of Co^4+^, which was essential for the OER to occur. Meanwhile, novel functional nanostructures have been explored as electrodes to tune their properties and improve electrocatalytic performances. A comparative study of Ru, Ir and Pt nanoparticles and their bulk materials on the electrocatalytic OER was examined.^124^ It was found that the intrinsic OER activities for nanoparticle catalysts decreased in the order of Ru > Ir > Pt. Ru nanoparticles showed outstanding OER performance but its practical application was limited by its stability problem while Pt nanoparticles suffered from additional deactivation compared to its bulk catalyst. Lee et al. studied the OER activities of noble metal oxide electrocatalysts such as RuO_2_ and IrO_2_ nanoparticles in acid and alkaline solutions.[[qv: 126a]] For the two electrocatalysts, RuO_2_ nanoparticles were found to have slightly higher intrinsic OER activities than IrO_2_ in both acid and basic solutions. In another study, the particle size effect on the electrocatalytic behaviour was also investigated using nanocrystalline RuO_2_.^144^ It showed that the polygonal prismatic nanocrystals of <20 nm in size showed an enhanced activity toward OER. This was attributed to that the smaller nanocrystals exhibited (110) and (100) oriented faces while the larger nanocrystals exhibited an additional (140) oriented face accompanying a decline in oxygen evolution activity. The (110)‐(100) face edge is thus thought to be a preferential site for OER, which is a desirable outcome from nanocrystal synthesis. Besides the facet‐dependent catalytic activities, amorphous Ru and its oxides demonstrated an increased OER performance in comparison to the crystalline phase (**Figure** **10**).^125^, ^145^ This was attributed to the higher concentration of coordinatively unsaturated sites in amorphous Ru, which facilitated the adsorption of reactants easier than the crystalline counterpart. Likewise, RuO_2_ supported on Sb‐doped SnO_2_ nanoparticles also exhibited greater activity for OER than RuO_2_ nanoparticles alone.^146^ The Sb‐doped SnO_2_ nanoparticles also reduced the usage of noble metals. It was reported that IrO_2_‐RuO_2_ supported on Sb‐doped SnO_2_ nanoparticles exhibited similar performance as IrO_2_‐RuO_2_ nanoparticles with a loading of 20 wt%.[[qv: 126b]]

Apart from the noble metal‐based electrocatalysts, recently earth‐abundant electrocatalysts have attracted much attention in water oxidation reaction.^130^, ^147^ Ni‐doped Ni_x_Co_3–x_O_4_ nanowire arrays with large surface area and efficient charge transfer have been demonstrated to be superior over their nanoparticle film equivalents in electrocatalytic OER.[[qv: 147b]] Amorphous binary and ternary metal oxides films of Fe, Co, and Ni with various metal composition were studied for electrocatalytic water oxidation.^130^, ^148^ It was found that a small amount of Fe in the mixed oxide matrix (Fe_100−y−z_Co_y_Ni_z_O_x_) produced a significant improvement in Tafel slopes while exceeding Fe concentration of >40% induced a detrimental effect. The binary phases of Co and Ni did not produce optimal catalytic behavior and the best catalytic performance was obtained for a film with composition at Fe_20_Ni_80_.[[qv: 130b]] In a similar report, the best amorphous electrocatalyst among the Fe‐Ni‐O_x_ series was found to be Fe_6_Ni_10_O_x_.^148^

*Hydrogen Evolution Reaction (HER)*: Pt is the most ideal HER electrocatalyst that requires a very low overpotential to generate a large cathodic current density.^149^ With high cost, it is less competitive in practical application. MoS_2_ becomes an alternative of active HER electrocatalysts^135^, ^150^ since the first report by Hinnemann et al. in 2005.^151^ Both experimental and computational results identified that the sulfur edges of MoS_2_ are the active sites for HER, which are structurally distinct from the inactive bulk material. As a result, there are more research focuses on optimising the edge sites of MoS_2_ nanostructures. Interestingly, amorphous MoS_2_ nanostructures lacking of well‐defined edge sites exhibited better electrocatalytic activity than the crystalline counterpart, which is probably attributed to the abundant of defect sites in the amorphous structure.[[qv: 150b,152]] To improve the electrocatalytic performance of MoS_2_, tremendous efforts have been made to engineer the electrocatalyst with more number of exposed edges as active sites. However, the poor intrinsic conductivity as a cathode material severely suppresses charge transport and thus the electrocatalysis efficiency.

Through synergistic structural and electronic modulations, controllable disorder engineering and simultaneous oxygen incorporation into MoS_2_ electrocatalysts were achieved to effectively improve HER performance. The short‐range disordered structure offered abundant unsaturated sulfur atoms as catalytically active sites for HER, and simultaneously the electronic structure induced by oxygen incorporation further improved the intrinsic conductivity.[[qv: 152b]] Besides, the direct growth of MoS_2_ and WS_2_ on carbon with vertically oriented nanosheet layers was reported to increase the active edge sites, overcome the limited electron/proton transport and facilitate fast release of small gas bubbles to maintain a large working area.[[qv: 135b]] The low bubble adhesion surface was also demonstrated from oriented nanostructured superaerophobic MoS_2_ film,^153^ which reduce gas bubble adhesion to promote their evolution/release from catalyst surface so as to maintain a constant working area and enhance the electrolyte contact with catalyst.[[qv: 129a,140,153]] On the other hand, the integration of MoS_2_ nanoparticles with graphene led to a strong synergistic effect resulting from the highly exposed edges of MoS_2_ and excellent electrical properties of graphene.[[qv: 150a]] The HER electrocatalysts showed excellent HER activities with a small overpotential. Meanwhile, analogous structures such as WS_2_[[qv: 135b,154]] also provoked tremendous interest as HER electrocatalysts for water splitting. Besides, other oxides such as MoO_3–x_,^132^ WO_2_ 133 and WO_3_ 134 also exhibited promising performance toward HER. There are other molybdenum‐based nanostructures that were studied as HER electrocatalysts for water splitting, including MoB,^155^ Mo_2_C,^155^, ^156^ NiMoN_x_,^157^ and Co_0.6_Mo_1.4_N_2_.^158^

Further, a series of first‐row transition metal dichalcogenides (MX_2_, M = Fe, Co, Ni; X = S, Se) were studied as HER electrocatalyst.^159^ The electrochemical studies showed that the optimal Tafel slope of approximately 40 mV per decade were achieved in CoS_2_, CoSe_2_ and NiS_2_, indicating the fast kinetics to drive the HER among many others.[[qv: 159a]] Meanwhile, metal phosphide nanostructures[[qv: 136d,160]] also expanded the family of HER electrocatalysts, particularly CoP,[[qv: 136a‐c,161]] Co_2_P^137^ and Ni_2_P.[[qv: 137a,138]] The metal phosphide nanostructures with controllable size, morphology, stoichiometry and structure are very appealing when considering that their physicochemical properties vary accordingly and thus influencing their electrocatalytic performance. For instance, the electrocatalytic properties of CoP nanostructures with different morphologies, including nanowires, nanosheets and nanoparticles were tested towards HER, and the best electrocatalytic activity and stability were obtained from CoP nanowires.[[qv: 136a]] The high electrocatalytic performance was attributed to the small charge transfer resistance in CoP nanowires, which promoted fast electrode kinetics while the catalyst stability was ascribed to the facile stacking of 1D CoP nanowires into a 3D macroporous film on current collector, which effectively facilitated bubble convection and prevented accumulating hydrogen bubbles on the surface of electrode and damaging the catalyst.

**Figure 11:**
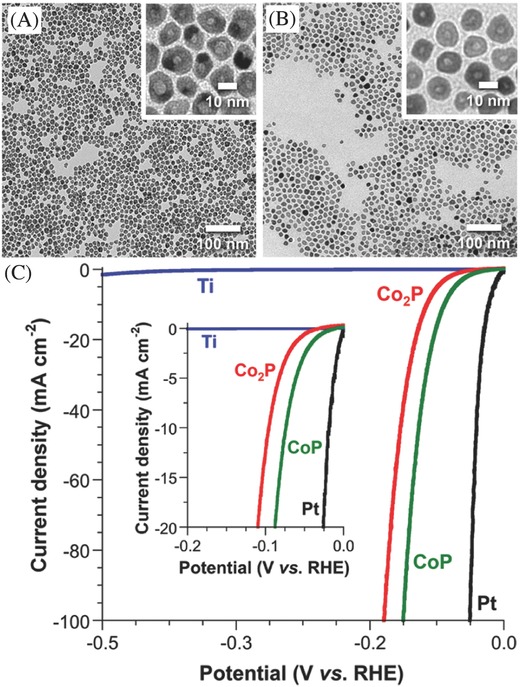
TEM images of (A) Co_2_P and (B) CoP nanoparticles, with enlarged regions in the insets. C) Polarization data (plots of current density vs. potential) in 0.5 M H_2_SO_4_ for Co_2_P/Ti and CoP/Ti electrodes, along with Pt mesh and bare Ti foil for comparison. The main plot shows an expanded region from 0 to −100 mA cm^−2^ and −0.5 to 0 V, while the inset shows an enlarged region from 0 to −20 mA cm^−2^ and −0.2 to 0 V. Reproduced with permission.[[qv: 137c]] Copyright 2015, ACS.

Meanwhile, the electrocatalytic of CoP was also compared to Co_2_P towards HER.[[qv: 137c]] Both CoP and Co_2_P are highly crystalline and have statistically indistinguishable size, dispersibility, faceting and overall morphological features to allow direct comparison on their HER activities based on their composition and structure (**Figure** **11**). The electrochemical analysis revealed that the HER activities increased with increasing phosphorus content (Co < Co_2_P < CoP) while Co_2_P required a slightly higher overpotential relative to CoP to drive HER. This suggested that CoP with a larger Co−P bond length offered a higher density of possible active sites that required proximal cobalt and phosphorus atoms on the surface.[[qv: 137c]] In another study, the HER activity of Co_2_P was compared to that of Co_1.33_Ni_0.67_P and Ni_2_P.[[qv: 137a]] Among the three electrocatalysts with rod nanostructures, Co_2_P exhibited the highest HER activity, indicating the importance of Co species in catalysing HER in water splitting.

## 6 Photoelectrochemical Water Splitting

For photoelectrochemical (PEC) water splitting, water is broken down by electrical charges (i.e. electron‐hole pairs) in catalysts upon light irradiation, which involves the electrochemical splitting of water into hydrogen and oxygen using the electrons and holes photogenerated, respectively. The first PEC water splitting was accomplished by Honda and Fujishima in the 1970s using TiO_2_ as photoanode and Pt as counter electrode.^162^ Since then, great attention has been received to develop various semiconducting metal oxides as photoanodes for PEC water splitting owing to their resistance to photocorrosion and cost effectiveness. Most of them are large band gap metal oxides that require excitation under ultraviolet light for PEC water splitting. In comparison, visible light‐responsive oxides (e.g. WO_3_, Fe_2_O_3_, and BiVO_4_) are unable to perform water reduction to produce hydrogen from water due to their low conduction band. This shortcoming requires an external bias between the photoanode and counter electrode so as to enable the oxide semiconductors to be used for PEC water splitting.

Despite its large band gap, nanostructured TiO_2_ is one of the mostly studied PEC photoanode in the forms of nanowires,[[qv: 36b,163]] nanotubes,^164^ nanorods,^165^ and nanoflowers,[[qv: 164b]] owing to their enhancement in PEC performance. With a narrow band gap of ≈2.6 eV, WO_3_ was recognized as an efficient visible light‐driven photoanode in PEC water splitting because of its ability to capture a sizeable fraction of solar spectrum, possess good photostability in acidic environments, and moderate hole diffusion length (≈150 nm). Morphological modifications and nanostructuring of WO_3_ are employed to increase the PEC performance by either improving the charge transport property or enhancing the photogeneration of charges. Jiao et al. investigated the morphology‐tailored WO_3_ as photoanode for PEC water splitting.^166^ Various sheet‐, wedge‐ and plate‐like nanostructures were directly grown into thin films on transparent conductive glasses using different capping agents, and the highest photoconversion efficiency of ≈0.3% were obtained from the sheet‐like film under simulated solar illumination. Other morphology‐controlled WO_3_ photoanodes such as nanorods and nanoplates were also reported to be active under simulated solar illumination.^167^

**Figure 12:**
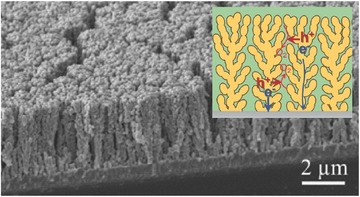
SEM image of the tree‐like nanoporous WO_3_ photoanode. Inset showing schematic illustration of charge transport/transfer processes in WO_3_ photoanodes. The hierarchical nanoporous WO_3_ photoanode has enhanced charge transport (or separation) and transfer efficiencies due to the open structure as well as partial orientation alignment. Reproduced with permission.^169^ Copyright 2015, RSC.

Recently, the vertically aligned WO_3_ nanostructured arrays have received great interest as a promising photoelectrode because of their superior PEC properties compared to those films with crystalline nanoparticles.^168^ This is attributed to the enhancement of charge transport properties resulting from the direct charge‐diffusion pathways found in vertically aligned nanostructured arrays. In comparison, the nanoparticle films have numerous grain boundaries which increase the interfacial charge recombination and thus retard the electron transfer to the back‐contacted conductive substrate. In another study, hierarchically organized and densely interconnected WO_3_ nanocrystals as photoanode greatly improved the photocurrent density, which was 9 times higher than that of a dense WO_3_ photoanode under simulated solar illumination (**Figure** **12**).^169^ This superior photocatalytic performance resulted from its nanoporous feature that greatly improved charge transport properties as well as allowed easy permeation of electrolyte into photoanode, thereby facilitating a shorter hole diffusion distance to electrolyte and efficient electron/hole separation. Band gap engineering via doping cations or anions into WO_3_ was used to tailor the electronic structure for improving PEC properties. A variety of dopants such as cations (e.g. Mg, Mo, Cr, Ti, Zr, Hf)^170^ or anions (e.g. C, N, S)[[qv: 170d,171]] were investigated to improve the photocatalytic properties of WO_3_, either by shifting the conduction band edge upward to a level above the hydrogen reduction potential or narrowing the band gap for visible light‐driven enhancement in photoactivity. Similarly, the integration of WO_3_ with plasmonic metals such as Au has been recognized as an effective strategy to improve PEC water splitting performance because of the enhanced light absorption, faster electron transport and higher hole injection yield resulting from surface plasmonic resonance effect of Au nanoparticles.[[qv: 167c]]

Another promising photoanode for water oxidation reaction and visible light‐responsivity is hematite, α‐Fe_2_O_3_, which possesses a small band gap of 2–2.2 eV and thereby permits significant visible light absorption up to 550–600 nm. Despite its ability to absorb visible light and chemical stability, the PEC activity of hematite is crucially limited by its poor electrical conductivity, short hole diffusion length (2–4 nm), high charge recombination rate, and poor OER kinetics. To overcome these shortcomings, a number of processes are known to improve the performance of hematite photoanode towards water splitting such as nanostructuring, doping and surface engineering to tailor their structural, electronic and optical properties. The poor electrical conducting property of hematite can be improved using elemental doping to increase donor density and further improve the electronic conductivity. A number of metal ions (e.g. Ti, Zr, Sn, Cr, Mo, Si) were doped into hematite as photoanodes for water splitting reaction.^172^ For instance, Ti‐doped hematite nanoparticles were reported to lower the onset potential and significantly improve the photocurrent density compare to the un‐doped hematite.[[qv: 172g]] The enhanced photocurrent was attributed to the improved donor density and reduced electron hole recombination induced by doping with Ti. Similarly, the enhanced PEC performance in Zr‐doped hematite was explained by a reduced electron‐hole recombination, mainly due to the increased electrical conductivity as a result of Zr doping.[[qv: 172c]] Sn dopant also served as an electron donor to increase the carrier density for improving electrical conductivity of hematite nanostructures.[[qv: 172d]] The effect of doping not only yielded noticeable increase in electrical conductivity, but also altered the surface electronic structure of hematite, which likely contributed to the improved PEC performance by passivating the surface trap states.[[qv: 172a,h]] On the other hand, the slow water oxidation reaction kinetics of hematite photoanode can be addressed by surface modification with cocatalysts (e.g. IrO_2_, Co_3_O_4_, Co‐Pi, Ni(OH)_2_).^173^ For instance, the most effective water oxidation cocatalyst, IrO_2_ nanoparticles were coupled to the surface of hematite to significantly reduce the onset potential by 0.2 V.[[qv: 173b]] Likewise, Co_3_O_4_, Co‐Pi and Ni(OH)_2_ prepared by electrodeposition or atomic layer deposition on hematite were developed as cheaper substitutes to noble metal oxides to reduce the overpotential of hematite photoanodes for water oxidation reaction.[[qv: 173a,c‐f]] Alternatively, surface engineering with a thin layer of metal oxides such as Al_2_O_3_, Ga_2_O_3_, In_2_O_3_, ZnO^174^ or Ag^175^ on hematite photoanodes is an effective way to improve water oxidation reaction by facilitating interfacial electron transfer, increasing photocurrent and reducing the onset potential for oxygen generation from water.

In addition to simple oxides, mixed metal oxides such as BiVO_4_ have attracted broad interest as water oxidation photocatalysts under visible light illumination.^176^ There are three crystalline phases of BiVO_4_ including monoclinic scheelite, tetragonal scheelite and tetragonal zircon structures, with band gaps of 2.4, 2.34, and 2.9 eV, respectively.^177^ The crystalline phases have strong influence on the photocatalytic properties of BiVO_4_, particularly, monoclinic scheelite was reported to exhibit higher photocatalytic activity for OER than the two other phases.^178^ However, the pure BiVO_4_ photoelectrode is limited by its poor photocurrent stability and low incident photon to current conversion efficiency at lower potentials, therefore high voltage is required to obtain a fairly high conversion efficiency, which may be associated with the high surface recombination of photogenerated electrons and holes. Like other photoanodes, doping and surface modification were used to overcome these limitations. For instance, the doping of W into BiVO_4_ increased the conductivity and carrier density and thus improve PEC performance than the undoped BiVO_4_.^179^ It was also reported that the doping of 3% Mo into BiVO_4_ significantly improved the photocurrent due to the enhanced conductivity and a possible increased hole diffusion length.^180^ The co‐doping of Mo and W into BiVO_4_ showed a water oxidation photocurrent that was more than 10 times higher than that of the undoped BiVO_4_.^181^ The active role of W and Mo facilitated the separation of excited electron‐hole pairs in BiVO_4_, thus leading to the enhanced photocatalytic performance. On the other hand, surface modification with a cocatalyst such as CoPi on BiVO_4_ electrode is an effective way to lower the activation energy and decrease the overpotential for water oxidation.^182^ Particularly, the PEC efficiency was enhanced 15 and 20 folds by CoPi in terms of OER and current generation, respectively.^183^ In this study, CoPi was suggested as a hole‐conducting electrocatalyst to allow the photogenerated electrons more mobile for consequently increasing conductivity to boost the PEC water oxidation performance of BiVO_4_. Also, the modification of BiVO_4_ electrode with Co_3_O_4_ improved the photoinduced charge separation efficiency and stabilized the photocurrent.^184^ Meanwhile, Co‐borate^185^ and Ni‐borate^186^ were demonstrated to successfully co‐catalyze the PEC water oxidation of BiVO_4_ electrode. In overall, the cocatalyst modification on BiVO_4_ electrode can clearly reduce the water oxidation overpotential, promote the charge transfer across the semiconductor–electrolyte interface, enhance the stability of BiVO_4_ photoanode, and thus result in higher PEC water splitting activity.

## 7 Photovoltaic‐Integrated Photoelectrochemical Water Splitting

**Figure 13:**
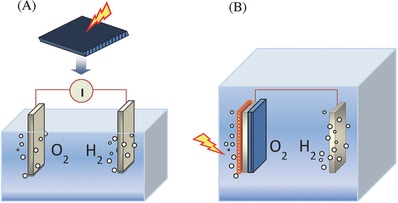
Schematic diagrams for the photovoltaic‐driven water‐splitting systems. A) Photovoltaic system and external electrolyzer. B) Photovoltaic‐integrated solar‐driven water splitting device.

Solar radiation can be converted to electrical energy through a photovoltaic system,^187^ which can be employed to generate electrical power for driving an external electrolyzer to produce hydrogen (**Figure** **13**A). Considering a crystalline silicon photovoltaic system with 18% efficiency coupled with an electrolyzer with 80% efficiency, the combined solar‐driven electrolyzer system yields a solar‐to‐hydrogen efficiency of ≈14%,^188^ which can be further increased theoretically when coupled to a photovoltaic system with higher efficiency. With separated construction of solar cell and electrolyzer, the solar cell does not required immersion into electrolyte and thus not susceptible to corrosion. The photovoltaic‐integrated solar‐driven water splitting directly utilizes renewable source of solar energy and does not emit greenhouse gases during hydrogen production. Although it is highly durable, the problem with this system also involves high production and installation cost of solar photovoltaics. This limitation is alleviated by integrating a photovoltaic device with an electrolyzer into a single device (Figure 13B), which reduces the cost and mechanical hurdles with separate construction and interconnection of solar and electrochemical cells.

Typically, such an integrated water splitting device comprises of light absorbers made up with single or multiple junctions. For instance, tandem configurations (multiple junctions) are conceived with the incorporation of light absorbers with different band gaps, stacked on top of each other, therefore increasing photopotential and utilizing a larger part of the solar spectrum. Another way is to interconnect several single‐junction cells in series which allows the efficient use of narrow band gap absorbers for better solar light absorption while still contributing the necessary photopotential to drive water splitting. These systems can increase the photopotential generated with a solar absorption across a broader spectrum, achieving higher solar‐to‐hydrogen efficiencies. In an early example, Khaselev and Turner pioneered monolithic tandem photovoltaic‐PEC cells using GaAs and GaInP_2_ to achieve a benchmark 12.4% solar‐to‐hydrogen efficiency.^189^ Later, Bradley et al. modified the surface of GaInP_2_ with phosphonic acids to shift the band edge alignment, closer to the desired overlap with the water redox potentials. The modification showed an improvement in band edge energetics and photocurrent onset of GaInP_2_.^190^ Peharz et al. also demonstrated an integrated approach for solar water splitting based on GaInP/GaInAs connected to a PEM electrolyzer and solar concentrator with solar‐to‐hydrogen efficiency of 18%.^191^ Despite high efficiencies, GaAs and GaInP_2_ are not ideal for large‐scale production, due to the high cost and scarcity of their components.

**Figure 14:**
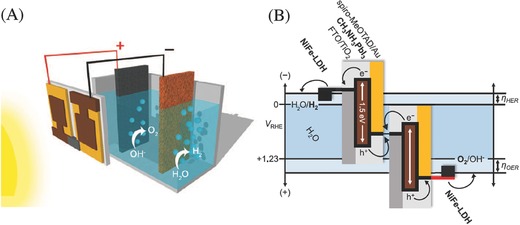
A) Schematic diagram of the water‐splitting device. B) A generalized energy schematic of the perovskite tandem cell with NiFe layered double hydroxides/Ni foam electrodes for water splitting. Reproduced with permission.^128^ Copyright 2014, AAAS.

Other recent advances also include solar water‐splitting device based on the combination of W‐doped BiVO_4_ photoanode and a two‐junction silicon solar cell with a ≈4.9% solar‐to‐hydrogen efficiency.^192^ The most notable work on “artificial leaf” involved the use of a triple junction and amorphous silicon photovoltaic interfaced to hydrogen‐ and oxygen‐evolving catalyst for yielding 4.7% efficiency.^193^ Jacobsson et al. reported a monolithic device for solar water splitting based on series interconnected CuIn_x_Ga_1–x_Se_2_ reaching over 10% solar‐to‐hydrogen efficiency.^194^ Grätzel et al. combined a perovskite tandem solar cell with a bifunctional earth‐abundant NiFe layered double hydroxide catalyst electrode to achieve a solar‐to‐hydrogen efficiency of 12.3% (**Figure** **14**).^128^ Likewise, smaller band gap materials are generally unstable to photocorrosion in aqueous solution. Moreover, highly acidic or highly alkaline electrolyte is usually used to lower the overpotential with an enhanced chemical corrosion. To ensure a stable water splitting operation, the fragile photoabsorbers has to be physically protected from corrosive electrolyte by coating or passivation of a stable integrated PEC device in tandem configuration in spontaneous solar water splitting.

**Figure 15:**
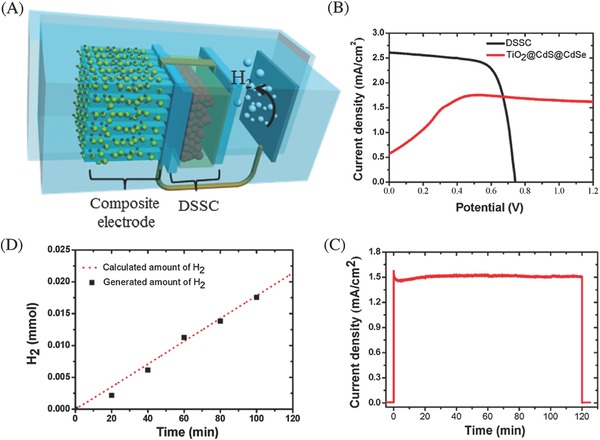
A) Schematic diagram of the dye‐sensitized solar cell tandem device with TiO_2_ nanotube arrays@CdS@CdSe composite electrode for hydrogen generation. B) J−V curves of the TiO_2_ nanotube arrays@CdS@CdSe composite electrode and dye‐sensitized solar cell in the two‐electrode system, C) J−t curve of the tandem cell, and D) calculated and generated amount of hydrogen gas from the tandem cell. Reproduced with permission.[[qv: 195c]] Copyright 2015, Elsevier.

Research efforts have recently focussed on the development of promising transition metal oxides such as TiO_2_, WO_3_, Fe_2_O_3_ and BiVO_4_ as photoelectrode in tandem water splitting device.^192^, ^195^ The envisioned strategy is to develop PEC tandem configurations based on a front visible light‐absorbing metal oxide photoelectrode combined with a rear small band gap solar cell. In these tandem configurations, the front photoelectrodes were composed of a material with a band gap of 2.2−2.5 eV, which are active under a limited solar spectrum. This causes a serious photocurrent mismatch with the rear solar cells. To increase the light harvesting of the front cell, a thicker photoelectrode could be adapted. However, such an approach would cause an abrupt decrease in the performance of the rear solar cells due to the poor transparency. Consequently, it remains challenging to synthesize a photoanode that exhibits not only a high photocurrent density but also a high transparency for tandem cells. Shin et al. designed high transparency front photoanodes using vertically aligned one‐dimensional TiO_2_ arrays on transparent conducting oxide which could maximize the light transmission properties for tandem configurations.[[qv: 195c]] For efficient visible light harvesting, CdS and CdSe composite particles were co‐deposited onto the TiO_2_ nanotubes in tandem with dye‐sensitized solar cells. Owing to the improved light transmission and PEC properties of the composite materials, the tandem device accomplished a solar‐to‐hydrogen efficiency of ≈2.1% in unassisted solar hydrogen generation (**Figure** **15**). Recently, optically transparent amorphous OER electrocatalyst, iron nickel oxide was deposited on high‐aspect ratio nanostructured hematite photoanodes in a perovskite tandem cell water splitting device. The low catalyst loading combined with its high activity at low overpotential resulted in significant improvement on the onset potential for PEC water oxidation and achieved solar‐to‐hydrogen conversion efficiencies in excess of 1.9%.^196^ In another study, Gurudayal et al. has demonstrated the use of a single organic‐inorganic halide perovskite solar cell to drive PEC water splitting with a solar‐to‐hydrogen efficiency of 2.4%. Stacking this with a Mn‐doped hematite photoanode has extended optical absorption and achieved high efficiency incorporating a single solar cell and hematite photoelectrode for cost effective water splitting.[[qv: 195d]] Shi et al. demonstrated a wireless monolithic tandem device composed of bipolar highly transparent BiVO_4_‐sensitised mesoporous WO_3_ films/Pt and a porphyrin‐dye‐based photoelectrode achieving 5.7% without any external bias.[[qv: 195e]] A sandwich infiltration process was used to produce a thin BiVO_4_ layer coated onto mesoporousWO_3_ films while preserving high transparency, enabling high photonic flux into the second dye‐sensitised photoanode.

## 8 Conclusions and Outlook

The demand of world energy is increasing but the major sources of energy still come from the Earth's reserves of fossil fuels particularly oil, coal and natural gas. Besides the commonly used steam reforming of natural gas, water splitting appears to be a very promising solution to produce hydrogen in the pursuit of carbon‐free and environmentally friendly energy. As the most available source on Earth and also the major resource of hydrogen, water is the main constituent in water splitting driven by different energy forms. Nowadays, water electrolysis has accounted for 4% of the world's hydrogen production. In the trend of recent research, there is a greater focus on solar energy driven hydrogen production. Through direct water splitting under sunlight, endless source of clean fuel can be produced for various applications. To improve the hydrogen generation efficiency, catalytic water splitting is emphasized in recent research including electrocatalysis, photocatalysis, thermochemical cycle and enzymatic reaction. Also, it is potentially important to convert biomass to hydrogen by biochemical energy via biological processes. A variety of sustainable hydrogen production pathways is not only limited to thermolysis, electrolysis, photolysis and biolysis of water, but also focus on their combinations including thermoelectrolysis, photoelectrolysis and biophotolysis of water.

In this review, we present the new advances in water splitting with focuses on photocatalytic and electrocatalytic hydrogen production. Many water splitting photocatalysts such as metal oxides with wide band gaps are successfully developed to be highly photoactive under ultraviolet light irradiation. One notable example is the highly efficient water splitting using NiO/NaTaO_3_:La photocatalyst with a maximum apparent quantum yield of 56% at 270 nm.^76^ As ultraviolet light accounts for only a small portion (4%) of solar energy, it is critical to develop more efficient photocatalysts under visible (53%) and infrared (43%) light irradiation so as to utilise more of the solar spectrum. However, only very low photocatalytic efficiencies were achieved until a recent work on visible light‐absorbing metal chalcogenides such as CdSe photocatalyst with a high efficiency of >36% at excitonic peak (520 nm) with the assistance of sacrificial reagent. Therefore, the narrow band gap semiconductor materials bring great potential to achieve high efficiency across a broader visible spectrum through band gap engineering by tuning size, doping metal/non‐metal elements or altering/reducing high conduction band position to modify the band structure of photocatalysts for enhancing photogenerated charge separation. When the visible light‐responsive metal oxides such as WO_3_, Fe_2_O_3_ and BiVO_4_ with low conduction band of >0, they are unable to perform water reduction to produce hydrogen from water. This shortcoming requires an external bias between the photoanode and counter electrode so as to enable the oxide semiconductors for use in PEC water splitting.

On the other hand, the electrolysis of water is used commercially for hydrogen production. In addition to the currently used noble metal‐based electrocatalysts, emerging non‐noble metal‐based electrocatalysts such as transition metal alloys, oxides, sulphides and phosphides (e.g. Ni‐Fe, Ni‐Fe‐Co, MoO_3–x_, WO_2_, WO_3_, MoS_2_, WS_2_, CoP, Co_2_P, Ni_2_P,) as well as bifunctional electrocatalysts (e.g. TiN@Ni_3_N, Ni_3_Se_2_/Ni, CoO/CoSe_2_, CoMnO@CN) have been developed for cost‐competitive water electrolysis. In addition to the direct use of electrical energy, a wide spectrum of solar radiation can be converted to electrical energy through a photovoltaic system for driving an external electrolyzer to produce hydrogen. Besides the separated construction and combined use of a solar cell with an electrolyzer, a photovoltaic device which comprised of light absorbers with different band gaps can be integrated with an electrolyzer into a single device to reduce the overall cost for hydrogen production when compared with coupled photovoltaic‐electrolysis systems. Although this technology is still in experimental stages, it already demonstrates promising efficiencies and reduced costs for hydrogen production.

As non‐renewable hydrogen production methods can only serve as a short‐term supply for the hydrogen economy, various renewable strategies are being developed to enable a hydrogen economy, which is expected to be realized as soon as possible before irreversible damage is done onto environment by a fossil fuel based economy. It is anticipated that the low cost, environmentally friendly photocatalytic water splitting at benign operating condition for hydrogen production will play an important role in the hydrogen production and contribute much to the coming hydrogen economy. In overall, solar driven hydrogen generation is ideal to produce green energy but it is an ongoing challenge to achieve this goal. Importantly, various factors such as electronic properties, chemical composition, structure, crystallinity, surface states and morphology of catalysts need be carefully considered to tune and determine their photocatalytic activity for drastically improving hydrogen production efficiency in practical applications.
